# A new H_2_S-specific near-infrared fluorescence-enhanced probe that can visualize the H_2_S level in colorectal cancer cells in mice†
†Electronic supplementary information (ESI) available: Experimental details, photophysical data, some fluorescence imaging figures, average fluorescence intensity figures and *in vivo* imaging. See DOI: 10.1039/c6sc05646f



**DOI:** 10.1039/c6sc05646f

**Published:** 2017-01-17

**Authors:** Kun Zhang, Jie Zhang, Zhen Xi, Lu-Yuan Li, Xiangxiang Gu, Qiang-Zhe Zhang, Long Yi

**Affiliations:** a State Key Laboratory of Medicinal Chemical Biology and College of Pharmacy , Tianjin Key Laboratory of Molecular Drug Research , Nankai University , Tianjin 300071 , China . Email: zhangqiangzhe@nankai.edu.cn; b State Key Laboratory of Organic-Inorganic Composites and Beijing Key Laboratory of Energy Environmental Catalysis , Beijing University of Chemical Technology (BUCT) , 15 Beisanhuan East Road, Chaoyang District , Beijing 100029 , China . Email: yilong@mail.buct.edu.cn; c State Key Laboratory of Elemento-Organic Chemistry , National Engineering Research Center of Pesticide (Tianjin) , Collaborative Innovation Center of Chemical Science and Engineering , Nankai University , Tianjin 300071 , China

## Abstract

Near-infrared (NIR) fluorescence-based sensors capable of selective detection of H_2_S *in vivo* would be useful tools to understand the mechanisms of diseases. A new NIR fluorescence probe **1** was developed for the detection of endogenous H_2_S in colorectal cancer cells in mice. **1** displayed an 87-fold fluorescence enhancement at 796 nm (with excitation at 730 nm) when reacted with H_2_S in a buffer (pH 7.4). **1** was water-soluble, cell-membrane-permeable, had low cytotoxicity and high selectivity and sensitivity for H_2_S. The properties of **1** enable its use in monitoring endogenous H_2_S in living cells, tissues, and mice. The bioimaging results indicated that (1) d-Cys could induce endogenous H_2_S production in living cells and stimulate angiogenesis; (2) tail intravenous injection of **1** into mice generated strong fluorescence in the liver while intraperitoneal injection of d-Cys could further enhance fluorescence in the liver *in vivo*; (3) importantly, endogenous H_2_S in colorectal cancer cells (HCT116, HT29) *in vitro* and in murine tumor models could be quickly and selectively detected by intratumoral injection of **1**. These results indicated that our new probe could serve as an efficient tool for the detection of cellular H_2_S in living animals and even for cancer diagnosis.

## Abstract 2

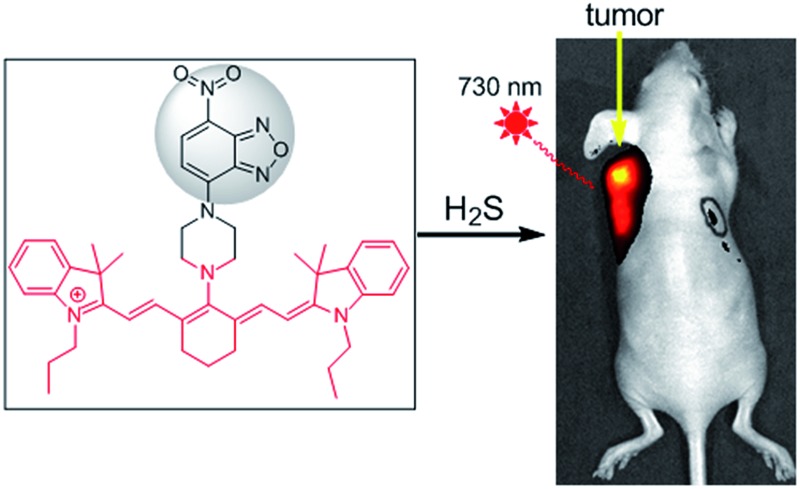
A highly sensitive H_2_S-specific near-infrared fluorescence-enhanced probe was developed for real-time imaging of endogenous H_2_S in colorectal cancer cells (HCT116 and HT29) in mice.

## Introduction

Hydrogen sulfide (H_2_S) has long been known as a toxic gas, however recent studies indicated that endogenously produced H_2_S has important physiological functions, and was also named as the third gasotransmitter after nitric oxide and carbon monoxide.^1^ Endogenous H_2_S can be enzymatically produced by three distinctive pathways including cystathionine β-synthase (CBS), cystathionine γ-lyase (CSE), and 3-mercaptopyruvate sulfurtransferase (3-MST) coupling with cysteine aminotransferase (CAT).^2^ Accumulating evidence suggests that H_2_S influences a wide range of physiological and pathological processes,^3–7^ including the modulation of blood vessel tone and cardioprotection,^3^ the endogenous stimulation of angiogenesis^4^ and mitochondrial bioenergetics.^5^ H_2_S inhibited nuclear factor-kB activation in oxidized low-density lipoprotein-stimulated macrophages.^6^ H_2_S also plays important roles in tumor biology, and it is suggested that both the inhibition of H_2_S biosynthesis and elevation of H_2_S concentration beyond a certain threshold could exert anticancer effects.^7,4b^ Nevertheless, the pharmacological character of H_2_S and the precise mechanisms by which H_2_S may be involved still remain largely unclear. Therefore, efficient tools for the visualization of endogenous H_2_S *in vivo* should be invaluable in further exploring H_2_S biology and even for the diagnosis of H_2_S-related diseases.

**Scheme 1:**
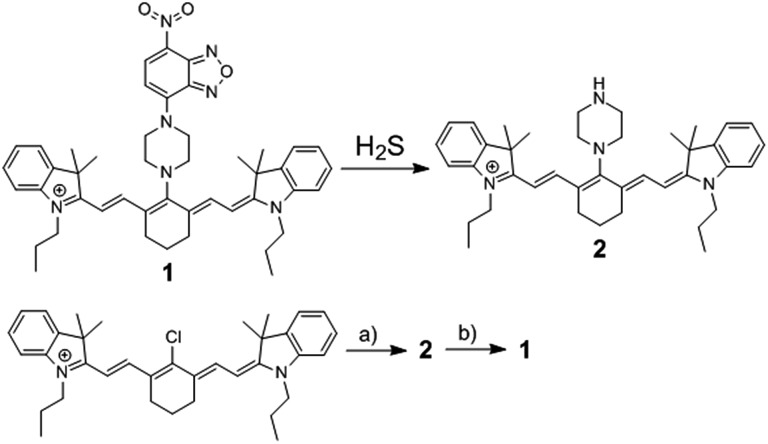
The chemical structure of fluorescence probe **1**, its reaction with H_2_S, and its synthetic routine. (a) Piperazine, 82.3%; (b) 7-nitro-1,2,3-benzoxadiazole chloride, 60.2%.

A fluorescence-based method has recently emerged as an efficient approach for the *in situ* and real-time detection of H_2_S in biological systems. Various fluorescence probes have been successfully employed to detect cellular H_2_S.^8–16^ However, H_2_S fluorescence probes for *in vivo* bioimaging are still rare,^8^ especially for the imaging of H_2_S-related diseases including cancers.^11*c*^ Suitable fluorescence probes for the imaging of H_2_S in tissues or individuals are preferred to meet certain requirements, such as a NIR optical window, enhanced fluorescence, fast and sensitive response, good selectivity, water-solubility and low cytotoxicity simultaneously. Organic reactions of nucleophilic additions are not suitable for the development of turn-on NIR probes due to the interruption of a large conjugate system by H_2_S addition.^10^ The reduction of a nitro group is relatively slow in a NIR probe,^12*c*^ while azide-based NIR probes are not employed for the imaging of H_2_S in animals.^12^ Thiolysis reactions should be one of ideal strategies for the development of NIR probes. Considering the concentration of GSH ranges from 1 to 15 mM depending on the cell types,^17^ we discovered a reaction of H_2_S-specific thiolysis of 7-nitro-1,2,3-benzoxadiazole (NBD) amines even in the presence of millimolar GSH,^16^ which was further employed herein for the development of a new NIR probe **1** (Scheme 1).

NIR light, especially at 700–900 nm, can improve the tissue depth penetration, offer low phototoxicity to cells and minimize the effect from background autofluorescence.^18^ Among NIR fluorochromes, cyanine dyes have excellent photophysical properties, outstanding biocompatibility, and low toxicity to living systems for the successful development of fluorescence probes.^19^ Herein we report a cyanine-based NIR probe **1** for the highly selective imaging of endogenous H_2_S in tissues and living mice. We also successfully applied probe **1** for monitoring intratumoral H_2_S by an *in vivo* fluorescence bioimaging technique, highlighting the potentially tumor-diagnostic value of this NIR probe.

## Results and discussion

### Synthesis and optical characterizations

The reaction of commercially available Cy7-Cl and piperazine in DMF provided **2**,^19*b*^ and the synthesis of **1** was easily achieved by coupling reaction of **2** and NBD-Cl (Scheme 1). A facile and economic synthesis is important for the wide use of such a probe. **1** was well characterized by ^1^H NMR, ^13^C NMR and HRMS.

Studies were carried out to use **1** for the detection of H_2_S under simulated physiological conditions (50 mM phosphate buffer saline (PBS), pH 7.4). The time-dependent absorbance (Fig. S1†) at NIR range for **1** exhibited a maximum at 700 nm, which shifted to around 740 nm after treatment with H_2_S. The NBD absorbance displayed a decrease at 450–500 nm and an increase at 565 nm, implying that H_2_S could cleave the NBD moiety from probe **1** to give **2**. This was further supported by the HRMS results of **1**, exhibiting a peak at *m*/*z* = 589.4268 after treatment with H_2_S, which corresponded to **2** ([M^+^] 589.4265). The absorbance of probe **1** at 700 nm exhibited a wide linear range in 2% DMSO-containing PBS (Fig. S2†), indicating that the probe had good water-solubility up to over 100 μM.

**Fig. 1:**
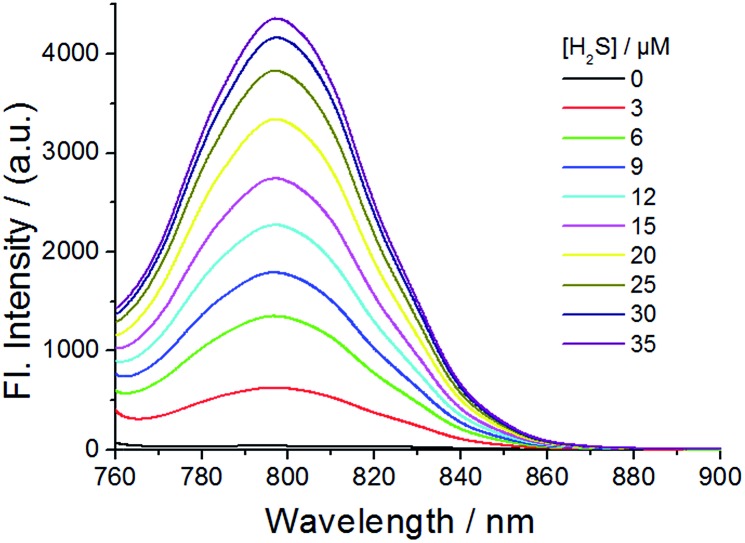
Emission spectra of probe **1** (10 μM) in the presence of various concentrations of H_2_S at 37 °C for 30 min in PBS buffer (50 mM, pH 7.4). Excitation, 730 nm.

**Fig. 2:**
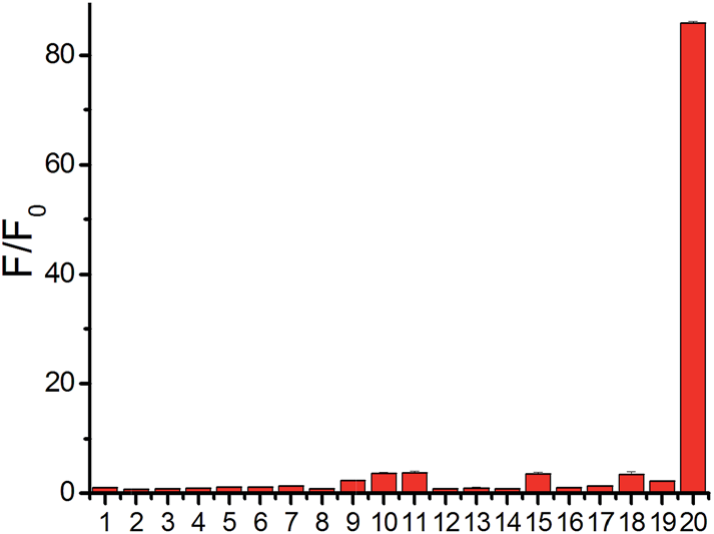
Relative fluorescence intensity at 796 nm of probe **1** (10 μM) with various species at 37 °C for 30 min in PBS buffer. Lane 1, only probe; lane 2, 100 μM Fe^3+^; lane 3, 100 μM Zn^2+^; lane 4, 100 μM H_2_O_2_; lane 5, 100 μM ClO^–^; lane 6, 100 μM NO_2_^–^; lane 7, 100 μM S_2_O_3_^2–^; lane 8, 100 μM SO_3_^2–^; lane 9, 1 mM Hcy; lane 10, 1 mM Cys; lane 11, 5 mM GSH; lane 12, 1 mM 2,2′-dithiodipyridine; lane 13, 1 mM 2,2′-dithiodipyridine + 100 μM H_2_S; lane 14, 1 mM cystamine; lane 15, 1 mM cystamine + 100 μM H_2_S; lane 16, 10 μM lysozyme; lane 17, 10 μM lysozyme + 100 μM dithiodipyridine + 50 μM H_2_S; lane 18, 10 μM BSA; lane 19, 10 μM BSA + 100 μM dithiodipyridine + 50 μM H_2_S; lane 20, 100 μM H_2_S.

The fluorescence of probe **1** was checked in the absence and presence of H_2_S in PBS buffer (pH 7.4) (Fig. 1, 2 and S3–S8†). The excitation spectrum of probe **1** in the presence of H_2_S indicated multiple peaks (689 nm, 714 nm, 733 nm and 764 nm) in the NIR range, providing flexibility for choosing excitation conditions in the NIR range. As shown in Fig. 1, the emission of probe **1** was nearly completely quenched at 796 nm. It is possible that electron transfer might occur from the NBD recognition unit to the cyanine group, resulting in quenching of the fluorophore, which is called the PET (photo-induced electron transfer) effect.^20^ After reacting with H_2_S at 37 °C, the NBD part is moved, and hence the PET effect disappears, resulting in the recovery of fluorescence emission of the NIR cyanine. The titration experiments indicated that a higher H_2_S concentration induced a larger turn-on fluorescence response (Fig. 1), with an off–on response up to 87-fold higher at 796 nm. Further data analysis revealed an excellent linear relationship (*r* = 0.994) between the fluorescence signal at 796 nm and the concentration of H_2_S (3–20 μM). We further titrated a low-millimolar concentration of H_2_S toward the probe **1** at 37 °C and determined a detection limit of 39.6 nM (Fig. S5†) by the 3*σ*/*k* method,^21^ indicating the high sensitivity of our probe. To obtain the reaction kinetics, the time-dependent fluorescence at 796 nm was recorded for data analysis (Fig. S6–S8†). The thiolysis rate for **1** by H_2_S was found to be up to 14.9 M^–1^ s^–1^ at 37 °C. These results implied that probe **1** could react with H_2_S both sensitively and efficiently.

A major challenge for H_2_S detection in biological systems is to develop a highly selective probe that exhibits a distinctive response to micromolar H_2_S concentrations over millimolar GSH concentrations. Probe **1** was incubated with various biological-related species in PBS buffer for 30 min and the maximum emission change at 796 nm was measured accordingly (Fig. 2). The tested species included reactive sulfur species (GSH, 5 mM; Cys, 1 mM; Hcy, 1 mM), reactive oxygen species (H_2_O_2_, ClO^–^), additional reactive sulfur species (SO_3_^2–^, S_2_O_3_^2–^), anions (NO_2_^–^), cations (Zn^2+^, Fe^3+^), low-molecular weight species and protein persulfides.^22^ The fluorescence intensity enhancement for any tested molecule in PBS buffer (pH 7.4) was very small except for H_2_S (Fig. 2), indicating the high selectivity of our NIR probe.^15^

### Fluorescence imaging of H_2_S in living cells by **1**

To test the biological applicability of the NIR probe, we firstly examined whether it can be used to detect H_2_S in living cells. Herein, mouse endothelial cell line (bEnd.3, with a low background endogenous H_2_S level) was employed for studying H_2_S-related angiogenesis.^4^ The results indicated that the **1**-treated cells (bEnd.3) showed weak fluorescence while the addition of both probe **1** and H_2_S to the cells resulted in obvious fluorescence, and a higher H_2_S concentration induced stronger fluorescence (Fig. S9†), implying that **1** could be used for the visualization of a change in H_2_S levels. The control experiments of aminooxyacetic acid (AOAA)-pretreated cells showed much weaker fluorescence than that of cells in the absence of an inhibitor (Fig. S10†), implying that **1** could be used for the detection of endogenous H_2_S in cells lines with a low background H_2_S level. Bright-field images show that cells retained a good morphology after incubation with **1**, suggesting good biocompatibility with the probe. The biocompatibility of **1** was further evaluated using cells by MTT assay (Fig. S11†). The results indicated that the probe possesses low cytotoxicity at the test concentration.

**Fig. 3:**
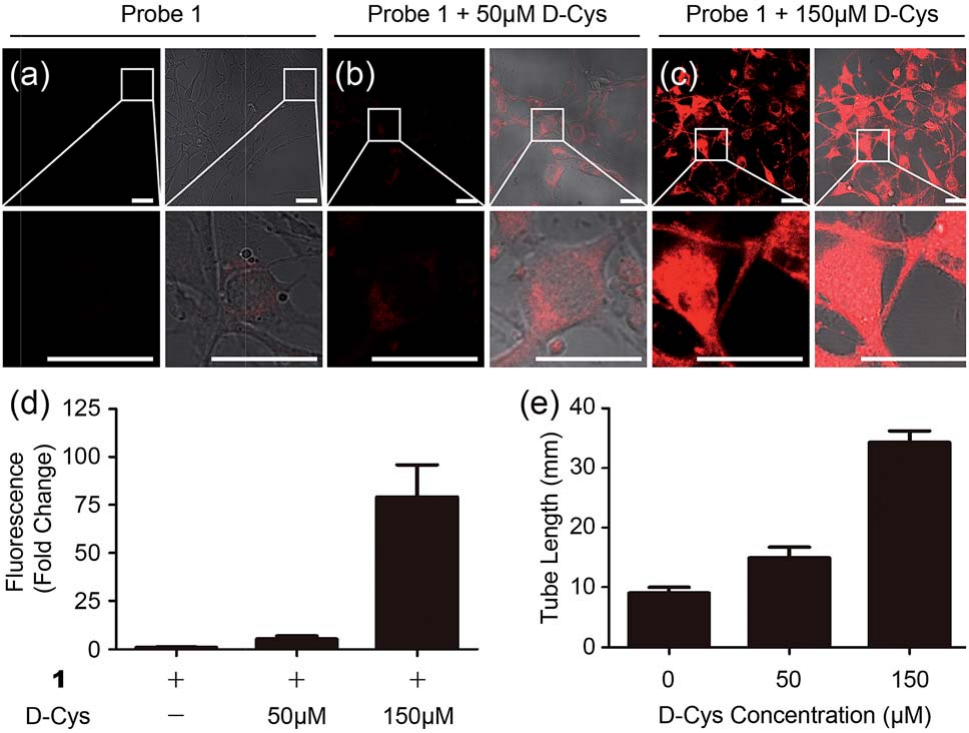
Microscope images of d-Cys-induced H_2_S in living cells and the angiogenesis assay in the presence of d-Cys. (a–c) Fluorescence images and overlap pictures of fluorescence and bright-field images of cells with 10 μM of probe **1**, with 50 μM of d-Cys and then 10 μM of probe, with 150 μM of d-Cys and then 10 μM of the probe, respectively. Scale bar, 25 μm. (d) The average fluorescence intensity of fluorescence images in panels (a–c). (e) Quantitative tube length at indicated d-Cys concentrations in Fig. S12.†

To test whether **1** could detect endogenous production of H_2_S in living cells, we employed d-Cys as a stimulant because our previous work indicated that d-Cys can induce enzymatic H_2_S production in mitochondria from HEK293 cells.^16*a*^ Cells with **1** displayed weak fluorescence, while in the d-Cys-stimulated cells strong fluorescence could be observed (Fig. 3a–c), implying that d-Cys induced endogenous H_2_S production. The average fluorescence of the images (Fig. 3d) implied that a higher concentration of d-Cys induced more H_2_S production in cells, and such an effect was further employed for the *in vitro* angiogenesis assay in the presence of d-Cys (Fig. S12†). The results indicated the tube length of the 50 μM d-Cys treated group became about 1.5-fold higher than that of the vehicle-treated group, while the 150 μM d-Cys treated group increased 2.5-fold (Fig. 3e). Therefore, d-Cys could induce endogenous H_2_S production in living cells and stimulate angiogenesis, which has not been observed previously. Taken together, these results indicated that probe **1** can be used for the study of endogenous H_2_S production and its biological functions.

### *In vivo* fluorescence imaging of H_2_S in mice by **1**

The high selectivity and excitation/emission wavelength of **1** in the NIR range shows it should be suitable for visualization of endogenous H_2_S *in vivo*. We firstly examined the suitability of **1** for visualizing exogenous H_2_S in mice. Mice were intraperitoneally (i.p.) cavity injected with probe **1** and Na_2_S, and then imaged using Xenogen IVIS Spectrum (IVIS Lumina II), a small animal *in vivo* imaging system with a 710 nm excitation filter and an ICG emission filter. The results (Fig. S13 and S14†) indicated that the mouse treated with both Na_2_S and the probe displayed much higher fluorescence than that treated only with the probe, and time-dependent images showed that the fluorescence response *in vivo* was fast with a *t*_1/2_ of less than 10 min, which is comparable with *in vitro* kinetic studies (Fig. S8†). The results indicated that the NIR probe possessed a desirable penetration depth for *in vivo* imaging, and could be further used to investigate endogenous H_2_S in living mice and tissues.

**Fig. 4:**
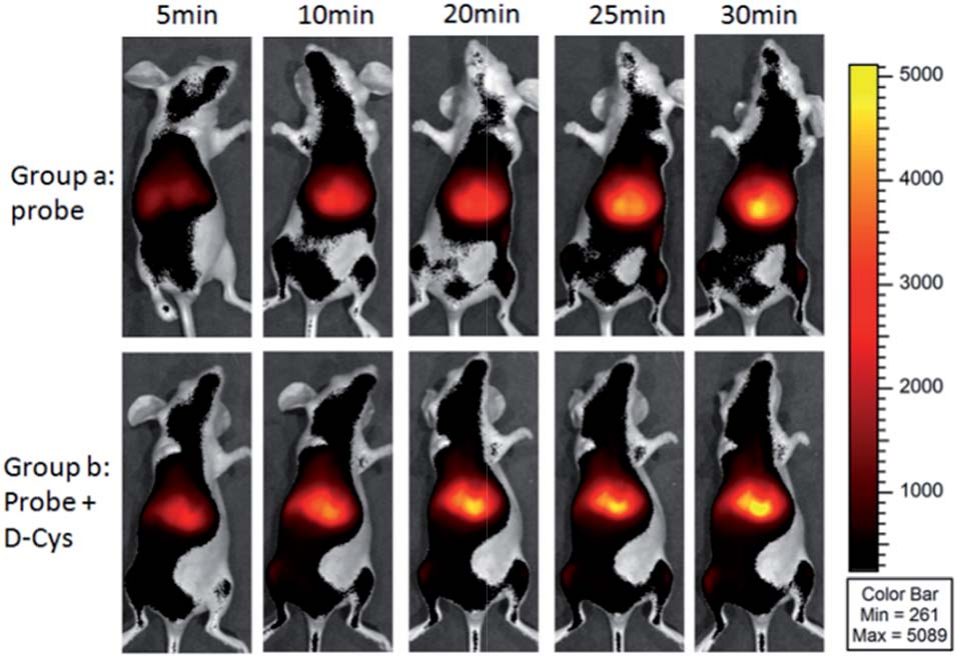
Representative fluorescence images for visualizing endogenous H_2_S with tail intravenous injection of probe **1** (150 μM, 200 μL) in living mice. For group a, time-dependent images of a mouse *via* only injection of probe **1**; for group b, time-dependent images of a mouse *via* i.p. injection of d-Cys, and after 30 min tail intravenous injection of probe **1**.

As shown in Fig. 4, the mice in group a were intravenously injected with probe **1** for 0–30 min, and intense time-dependent enhanced fluorescence could be observed in the liver of living mice. We also investigated the d-Cys-induced H_2_S in living mice with probe **1**. The mice in group b were i.p. injected with d-Cys. After 30 min, the mice were intravenously injected with probe **1**. A time-dependent fluorescence increase in the liver was also observed (Fig. 4). The average fluorescence of group b was about 10–20% higher than that of group a at various time points (Fig. S15†), implying that d-Cys could be metabolized into H_2_S in living mice and greatly occurred in the liver. These results were further confirmed by tissue imaging from the intravenous injection in mice (Fig. S16†). Fluorescence imaging indicated that H_2_S was produced in the tissues of the liver and the kidney, with the fluorescence intensity of the liver being larger than that of the kidney. While in the case of d-Cys-induced H_2_S, stronger fluorescence was observed for both liver and kidney tissues than that of the uninduced mice. The western blotting results indicated that the H_2_S-produced enzymes (*e.g.* CBS) in the liver are more expressed than that of the kidney (Fig. S16F†), which is consistent with our fluorescence imaging results. Similar results of endogenous H_2_S in the liver were also observed with the injection of a lower amount of probe **1** in another group of mice (Fig. S17 and S18†). These observations clearly demonstrate that **1** is an effective probe for endogenous H_2_S at the organism level.

### Fluorescence imaging of endogenous H_2_S in cancer cells and in mice by **1**

**Fig. 5:**
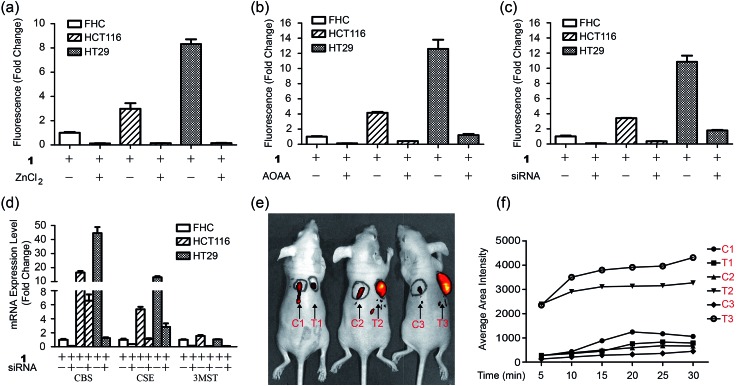
Fluorescence imaging of endogenous H_2_S in CBS-overexpressed cancer cells in mice. (a) Average fluorescence of images of living cells (FHC, HCT116, HT29) in the presence or absence of ZnCl_2_. (b) Average fluorescence of images of living cells in the presence or absence of inhibitor AOAA. (c) Average fluorescence of images of living cells in the presence or absence of siRNAs. (d) The mRNA expression level for H_2_S-produced enzymes in three cells lines in the presence or absence of siRNAs. (e) *In vivo* fluorescence images of mice with skin-pop (s.p.) or intratumoral injection of probe **1**. The left injection positions (C1, C2, C3) were for control purposes, while the right injection positions (T1, T2, T3) were for FHC, HCT116, and HT29 grafted cell positions. Observed tumors were formed in the HCT116 (T2) and HT29 (T3) xenograft mice. (f) The time-dependent average fluorescence of C1–C3, T1–T3 positions in mice in panel (e).

Importantly, we hope to use the NIR H_2_S probe to image H_2_S-related cancers *in vivo*. Herein human colorectal epithelial cancer cell lines HCT116 and HT29 were selected as model biological systems and human normal colorectal epithelial cell line FHC was used as a control.^7*c*^ We tested the fluorescence images of these cell lines with probe **1** (Fig. 5a and S19†). The results indicated that the **1**-treated cancer cells (HCT116 or HT29) showed stronger fluorescence than that of the FHC cells. The control experiments of ZnCl_2_-pretreated cells showed nearly no fluorescence for all cell lines. Changes of the endogenous H_2_S level in living cells by inhibitors AOAA or siRNA were further performed by the gene-knockdown-based fluorescence imaging strategy from our previous work.^11d,23^ Normal cells and specific gene-silencing cells were used for the bioimaging of endogenous H_2_S with probe **1** (Fig. S20†). The fluorescence of AOAA-treated or siRNA-treated cells was significantly weaker than that of the corresponding untreated cells (Fig. 5b and c). The mRNA expression level of H_2_S-produced enzymes of all cell lines and siRNA-silenced cells were checked by quantitative real-time PCR (Fig. 5d). The results indicated that both cancer cells (HCT116, HT29) expressed more H_2_S-produced enzymes, CBS and CSE, than that of the control cells (FHC), which explained the higher H_2_S level in cancer cells from our fluorescence imaging results. The expression of the H_2_S-produced enzymes could be downregulated *via* RNAi to give a low endogenous H_2_S level in living cancer cells, which could be visualized by our NIR probe.

Now that probe **1** could detect endogenous H_2_S in colorectal cancer cells, imaging of intratumoral H_2_S *in vivo* was further tested. FHC, HCT116, and HT29 cells were grafted into the nude mouse, respectively, to produce murine xenograft tumor models. After the direct skin-pop (s.p.) injection of **1** in PBS buffer into the tumors of HCT116- or HT29-xenograft mice, the emission was collected in the NIR range. Control experiments contained (1) the s.p. injection of **1** into other nontumoral parts of the same mice and (2) a FHC-xenograft mice (Fig. 5e). To kinetically observe the change in the fluorescence intensity, time-dependent experiments were performed (Fig. 5f, S21 and S22†). The results indicated that strong fluorescence in the tumor part could be observed quickly after intratumoral injection (5 min), while the control experiments only showed very weak fluorescence under the same conditions. Time-dependent fluorescence intensity measurements of these mice (Fig. 5d) indicated that the fluorescence of HCT116 and HT29 xenograft tumors is much higher than that of other parts (C1–C3, T1). Pre-injection of inhibitor AOAA significantly decreased the intratumoral fluorescence (Fig. S23†). Considering that H_2_S could be a biomarker in CBS- or CSE-overexpressed cancer cells (colorectal and ovarian cancers),^7*c*^ we proposed that our probe **1** combined with fluorescence bioimaging in the NIR range could provide a potential tool for the *in vivo* monitoring of endogenous H_2_S in the tumor. To our knowledge, this is the first time a NIR probe has been used for the monitoring of H_2_S in tumors *in vivo*.^24^

**Fig. 6:**
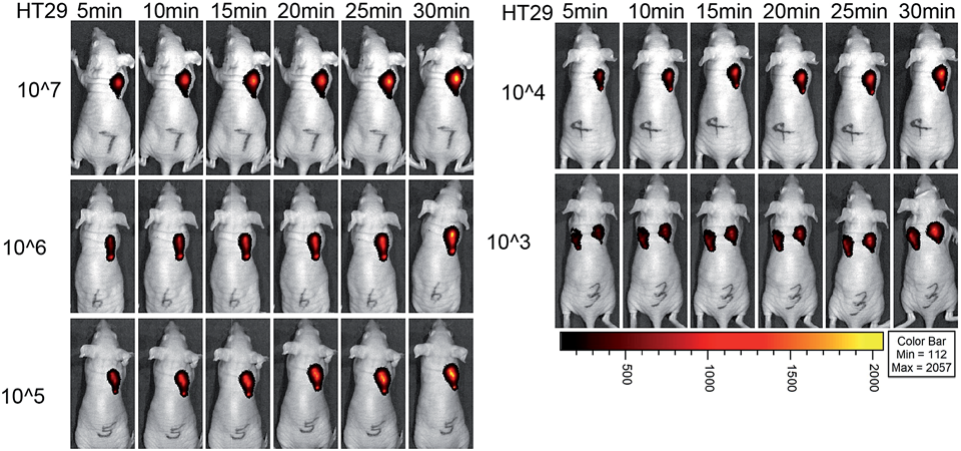
Cancer cell detection limit and sensitivity of the NIR probe in living mice. Fluorescence images of mice with injection of 10^7^, 10^6^, 10^5^, 10^4^ and 10^3^ HT29 cells at different time points. The control of only the PBS-injected position was in the left part of the mouse which was injected with 10^3^ HT29 cells in the right part.

Moreover, we used the NIR probe to test the detection limit of colorectal cancer cells *in situ* in living mice (Fig. 6). Mice were injected with different numbers of HT29 cells and the probe was used to image the cancer cells at 24 h after injection. A significant fluorescence signal was detected in the cell-injected region even at 5 min for 10^7^ cancer cells. After 30 min, a strong fluorescence signal could be observed for 10^3^ to 10^7^ HT29 cells in living mice, and the signal intensity for 10^3^ cells was stronger than that for the PBS control (Fig. S24†). Taking the results from the *in vitro* and *in vivo* experiments together, we believe that the NIR probe is highly sensitive for imaging endogenous H_2_S in cancer cells and in mice with an excellent detection limit.

## Conclusions

In summary, we have developed a new NIR fluorescence probe **1** capable of real-time imaging of endogenous H_2_S in cells, tissues (containing tumors) and mice. **1** is highly selective, has enhanced fluorescence (87-fold), a fast-response, is water soluble and has a low cytotoxicity with both an excitation and emission larger than 700 nm. The properties of **1** enable its use for noninvasive *in vivo* imaging of endogenous H_2_S without interfering with biological autofluorescence. Fluorescence imaging combined with the tail intravenous injection of **1** revealed that endogenous H_2_S heavily existed in the liver of living mice and d-Cys could induce more H_2_S production in the liver *in vivo*. Moreover, the endogenous H_2_S in colorectal cancer cells and in the tumors of mice could be detected by our probe tool. This new probe could serve as an efficient tool for the detection of cellular H_2_S in living animals and even for tumor diagnosis.
