# Supplementary material for: A new H2S-specific near-infrared fluorescence-enhanced probe that can visualize the H2S level in colorectal cancer cells in mice
Source: Chem Sci. 2017 Jan 17;8(4):2776–81. doi: 10.1039/c6sc05646f (PMC5426460; doi:10.1039/c6sc05646f)
Supplement: Supplementary file 1 [file SC-008-C6SC05646F-s001.pdf]

Electronic Supplementary Material (ESI) for Chemical Science.
This journal is © The Royal Society of Chemistry 2017

Supporting Information

A new H_2S-specific near-infrared fluorescence-enhanced probe can visualize H_2S level in colorectal cancer cells in mice

Kun Zhang,[‡a] Jie Zhang,[‡b] Zhen Xi,[ac] Lu-Yuan Li,[a] Xiangxiang Gu,[a] Qiang-Zhe Zhang,*[a] and Long Yi*[bc]

[a] State Key Laboratory of Medicinal Chemical Biology, College of Pharmacy and Tianjin Key Laboratory of Molecular Drug Research, Nankai University, Tianjin 300071, China.

[b] Beijing Key Lab of Bioprocess and State Key Laboratory of Organic-Inorganic Composites, Beijing University of Chemical Technology (BUCT), 15 Beisanhuan East Road, Chaoyang District, Beijing 100029, China.

[c] Collaborative Innovation Center of Chemical Science and Engineering, and State Key Laboratory of Elemento-Organic Chemistry, National Engineering Research Center of Pesticide (Tianjin), Nankai University, Tianjin 300071, China

‡ These authors contributed equally to this work.

Experimental part 2-8

Supporting figures 9-25

Experimental part

General chemicals and instruments. All chemicals and solvents used for synthesis were purchased from commercial suppliers and applied directly in the experiments without further purification. Merck silica gel 60 (100-200 mesh) was used for general column chromatography purification. ^{1}H NMR and ^{13}C NMR spectra were recorded on a Bruker 400 spectrometer, tetramethylsilane (TMS) or residual solvent peaks as internal standard (0 ppm) substances. High-resolution mass spectra (HRMS) were obtained on an Agilent 6540 UHD Accurate-Mass Q-TOFLC/MS or Varian 7.0 T FTICR-MS. The UV-visible spectra were recorded on a UV-3600 UV-VIS-NIR spectrophotometer (SHIMADZU, Japan). Fluorescence study was carried out using F-280 spectrophotometer (Tianjin GangdongSci& Tech., Development. Co., Ltd). For selective experiments, the persulfides were prepared according to a previous method (*ACS Chem. Biol.*, 2013, **8**, 1110-1116). 2,2'-Dithiodipyridine (1 mM) or cystamine (1 mM) in degassed PBS buffer (pH 7.4) was added freshly prepared sodium sulfide (100 μM) and the solution was incubated at room temperature for 3 h in sealed tube. The resulting low-molecular persulfides were directly used in fluorescent test without further purification. For protein persulfides, 20 μM BSA (bovine serum albumin) or lysozyme in degassed PBS buffer was incubated with 2,2'-dithiodipyridine (100 μM) at rt for 3 h, and purified by passing through a PD-10 column (GE Healthcare). The isolated protein (10 μM) was then incubated with Na_2S (50 μM final concentration) at rt for 1 h and purified by PD-10 column to give the protein persulfide (10 μM final concentration), which was immediately used in further test.

Synthesis of 2. 2 was prepared according to a previous literature (*J. Am. Chem. Soc.* **2014**, *136*, 5351). To a solution of IR-780 iodine (200.1 mg, 0.3 mmol) in anhydrous DMF (15 mL) under nitrogen atmosphere was added piperazine (103.4 mg, 1.2 mmol). After being stirred for 4 h at 85 °C, the mixture was cooled to room temperature. Removal of solvent under reduced pressure and purification by silica gel column chromatography with dichloromethane/methanol (40:1) as the eluent generated **2**

(176.8 mg, 82.3%) as a blue solid. ^{1}H NMR (400 MHz, DMSO-d_6) δ 7.60 (d, J = 13.5 Hz, 2H), 7.52 (d, J = 7.3 Hz, 2H), 7.36-7.30 (m, 2H), 7.28 (d, J = 7.8 Hz, 2H), 7.13 (t, J = 7.3 Hz, 2H), 5.99 (d, J = 13.6 Hz, 2H), 4.03 (t, J = 7.0 Hz, 4H), 3.71-3.64 (m, 4H), 3.22-3.15 (m, 4H), 2.49-2.45 (m, 4H), 1.76-1.65 (m, 6H), 1.62 (s, 12H), 0.92 (t, J = 7.4 Hz, 6H).

Synthesis of probe 1. To a solution of **2** (142.4 mg, 0.2 mmol) in anhydrous dichloromethane (10 mL) under a nitrogen atmosphere was added DIPEA (52 μL, 0.3 mmol). After stirring for 5 min, a solution of NBD-Cl (44.0 mg, 0.22 mmol) in dichloromethane (2 mL) was added dropwise at 0 °C. Further stirring for overnight at room temperature was followed by solvent removal under reduced pressure and purification by silica gel column chromatography with dichloromethane/methanol (200:3) as the eluent to generate **1** (106.0 mg, 60.2%) as a blue solid. ^{1}H NMR (400 MHz, DMSO-d_6) δ 8.60 (d, J = 9.0 Hz, 1H), 7.72 (d, J = 13.5 Hz, 2H), 7.45 (d, J = 7.3 Hz, 2H), 7.37 – 7.25 (m, 4H), 7.12 (t, J = 7.3 Hz, 2H), 6.84 (d, J = 9.2 Hz, 1H), 6.04 (d, J = 13.6 Hz, 2H), 4.48 - 4.38 (m, 4H), 4.06 (t, J = 7.0 Hz, 4H), 3.97 - 3.87 (m, 4H), 2.55 (t, J = 6.3 Hz, 4H), 1.84 – 1.68 (m, 6H), 1.58 (s, 12H), 0.95 (t, J = 7.4 Hz, 6H); ^{13}C NMR (101 MHz, DMSO-d_6) δ 170.2, 169.1, 145.1, 144.9, 144.8, 142.6, 140.6, 140.3, 136.3, 128.3, 124.3, 123.4, 122.1, 122.0, 110.2, 104.2, 97.0, 53.6, 52.8, 50.6, 47.8, 44.2, 41.9, 28.3, 24.7, 21.3, 20.0, 18.1, 16.7, 12.5, 11.2. HRMS (ESI): m/z 752.4253 $[M]^+$ (calcd for $C_{46}H_{54}N_7O_3^+$, 752.4283).

Cell culture. bEnd.3cells (mouse endothelial cell line) were grown in high glucose DMEM (GIBICO) supplemented with FBS (10%), penicillin (100 U mL^{-1}), streptomycin (100 UmL^{-1}), and L-glutamine (4 mM). HCT116 cells (human colorectal epithelial cancer cell line) were grown in McCoy's 5A with FBS (10%), penicillin (100 U mL^{-1}), streptomycin (100 U mL^{-1}). FHC (human normal colorectal epithelial cells line) and HT29 cells (human colorectal epithelial cancer cell line) were grown in RPMI 1640 supplemented with FBS (10%), penicillin (100U mL^{-1}), streptomycin

(100 U mL^{-1}). All cells were maintained in an incubator at 37 ℃with 5% CO_2/air environment.

Cytotoxicity assay. The *in vitro* cytotoxicity was measured using standard methyl thiazolyltetrazolium (MTT, Sigma-Aldrich) assay in bEnd.3 cell lines. Briefly, cells growing in log phase were seeded into 96 well cell-culture plate at 1 × 10^4/well. The probe **1** (100 µL/well) at concentrations of 0−33 µM was added to the wells of the treatment group, and 100 µL/well DMSO diluted in DMEM at final concentration of 0.5% to the negative control group, respectively. The cells were incubated for 0.5 and 1 h at 37 °C under 5% CO_2, respectively. The combined MTT/PBS solution was added to each well of the 96-well assay plate and incubated for an additional 4 h. An enzyme-linked immunesorbent assay (ELISA) reader (Bio-Rad Laboratories, Hercules, CA, USA) was used to measure the OD_{490} (absorbance value) of each well referenced at 490 nm. The following formula was used to calculate the viability of cell growth: viability (%T) = A1/A2 * 100%, where A1 denotes absorbance value of treatment group, and A2 denotes absorbance value of control.

Confocal fluorescence imaging for living cells. Cells were maintained in exponential growth phase, and then seeded in a glass-bottom 35 mm plate (~ 2×10^4 cells per well). The cells were excited by a 635 nm laser diode and detected at BA = 655−755 nm.

For the exogenous H_2S imaging, bEnd.3 cells were firstly treated with probe (10 µM) at 37 ℃ for 30 min, washed by PBS, and then incubated with Na_2S (150 and 450 µM) for 30 min. Control cells were treated with just probe. After being washed with phosphate-buffered saline (PBS) twice, the cells were imaged by using a confocal laser scanning microscope (Leica, Wetzlar, Germany).

For D-Cys-induced H_2S imaging, bEnd.3 cells were firstly treated with D-Cys (50 or 150 µM) at 37 ℃ for 20 min and then incubated with probe **1** (10 µM) for 30 min.

Control cells were treated with just probe. After being washed with PBS twice, the cells were imaged by using a confocal laser scanning microscope (Leica, Wetzlar, Germany).

For endogenous H_2S imaging, FHC, HCT116, HT29 cells were incubated with probe **1** (10 μM) for 30 min, and then washed with PBS twice before imaging. In the control experiments, three cells lines were incubated with 1 mM $ZnCl_2$ or 200 μM AOAA inhibitor for 30 min, washed by PBS twice, and then incubated with probe **1** (10 μM) for 30 min. After being washed with PBS twice, the cells were imaged as description above.

***In vitro* angiogenesis assay.** Forty-eight-well plates were coated with 40 μL of Matrigel (R&D Systems) and let to solidify for 30 min at 37 °C. bEnd.3 cells were seeded (3×10^4 cells/well) on top of the solidified Matrigel and treated with D-Cys (50 and 150 μM) or vehicle for 12 h. Formation of the capillary tubule structures was observed and digitally photographed well acquired at 4 × magnification (ECLIPSE Ti, Nikon, Tokyo, Japan). Tubule lengths and areas were quantified by using Image-Pro Plus 6.0 software (Media Cybernetics, Rockville, MD, USA).

***In vivo* fluorescence imaging experiments.** All experimental procedures involving animals were in accordance with the Guide for the Care and Use of Laboratory Animals (NIH publications nos. 80–23, revised 1996) and were performed according to the institutional ethical guidelines for animal experiment. The accreditation number of the laboratory is SYXK (Jin) 2014-00003 promulgated by Tianjin Science and Technology Commission.

For exogenous H_2S imaging, the female nude mice (6 weeks) were i.p. injected with probe **1** (150 μM, 200 μL) for 30 min. The mice were imaged on an IVIS Lumina II system (Xenogen/PerkinElmer), a small animal *in vivo* imaging system with a 710 nm excitation filter and an ICG emission filter. Next, Na_2S (150 μM, 200 μL) was injected into the intraperitoneal cavity, and then images were collected within 30 min.

For endogenous H_2S imaging, the peritoneal cavities of female nude mice (6 weeks) were injected with D-Cys (150 μM, 200 μL) and incubated for 30 min. While the control mice were used without D-Cys treatment. Next, the mice were tail intravenously injected with probe **1** (30 μM, 200 μL or 150 μM, 200 μL), and *in vivo* images were taken at indicated time intervals within 30 min.

Tissue fluorescence imaging. For organ imaging, the mice were euthanized and the organs were dissected after *in vivo* imaging experiments. The isolated liver and kidney tissues were imaged with the IVIS System.

Western blotting. Tissue samples were homogenized and subjected to SDS/PAGE. The proteins were transferred to a polyvinylidenedifluoride membrane (Roche Molecular Biochemicals, Quebec, Canada), blocked with 5% nonfat milk powder in TBS-T buffer (20 mM Tris-HCl, pH 7.4, 137 mM NaCl, and 0.1% Tween) for 1 hour at room temperature, incubated overnight at 4 °C with primary antibody against the target proteins and then incubated with appropriate HRP-conjugated secondary antibodies. The films were developed with the ECL System (Millipore, Billerica, MA, USA). CBS (#sc-67154, 1:1000), CSE (#sc-135203, 1:1000), 3MST (#sc-376168, 1:1000) were purchased from Santa Cruz Biotechnology (Santa Cruz, CA, USA).

Murine tumor model and fluorescence imaging. About 10^7 FHC, HCT116, and HT29 cells were grafted into the nude mouse, respectively. Tumors with volume of around 500 mm^3 were formed after 14 days. The xenograft tumors were intratumorly injected with probe **1** at a final concentration of 10 μM for tumor volume. Then the mice were imaged on an IVIS Lumina II system (Xenogen/PerkinElmer), a small animal in vivo imaging system with a 710 nm excitation filter and an ICG emission filter.

Detection of cancer cells in vivo. Mice were subcutaneously injected with 100 μL of HT29 cell suspensions with different concentrations (1×10^8, 1×10^7, 1×10^6, 1×10^5, 1×10^4 cells/ml PBS) or 100 μL of PBS buffer in the nude mouse, respectively. After 24 h of cell injection, the mice were injected with probe **1** (50 μL, 10 μM) in the position of cancer cells or PBS control. Optical imaging experiments were performed at 5, 10, 15, 20, 25, and 30 min after the injection of our NIR probe in the *in vivo* imaging system.

Quantitative real-time polymerase chain reaction. Cells were collected and homogenized in Trizol (Qiagen, Valencia, CA, USA), and RNA extraction and reverse transcription were performed according to the manufacturer's instructions. For PCR amplification of the cDNA fragment coding for targeted genes, the sense and antisense primer sequences for CBS, CSE，3MST were: 5'-GTGTG ATGGG AAGCT GGACA-3' and 5'-TTGTC TGCTC CGTCT GGTTC-3'; 5'-AAAGA CGCCT CCTCA CAAGG-3'and 5'-ATTCA AAACC CGAGT GCTGG-3'; 5'-CCGAG ACGGC ATTGA ACCT-3' and 5'-CCACC AGTGG CTTAG ACAGG-3', respectively.

RNAi experiments. Cells (FHC, HCT116, HT29) in exponential growth status were seeded in 12-well plates (approximate 8×10^4 /well) to reach about 70% confluence. The culture medium was changed into serum free OPTI-MEM I (GIBICO/Invitrogen, USA), 0.4 mL/well before transfection. Then, cells were co-transfected with siRNA sequences (Table S1) with Lipofectamine 2000 (Invitrogen, USA) (total transfection volume 0.5 mL). After 4 h incubation in 37 °C, OPTI-MEM I was changed into normal culture medium for further 20 h incubation. After that, cells were digested with 0.25% trypsin enzyme (GIBICO/Invitrogen, USA) and seeded in glass-bottom 35 mm plate with the density about 2×10^4/well. When cells began to stretch and confluence reached about 80-90%, confocal microscopy analysis was done

as described above. At the same time, total RNA was extracted from corresponding samples to perform the quantitative RT-PCR to confirm the gene-silencing efficiency.

Table S1. Sequences of siRNAs targeting difference enzymes involving in H_2S biogenesis.

CBS gene	5‘ GGAAGAAGUUCGGCCUGAA dTdT 3‘<br>3‘ dTdT CCUUCUUCAAGCCGGACUU 5‘
GeneID：875	5‘ GGAACUACAUGACCAAGUU dTdT 3‘<br>3‘ dTdT CCUUGAUGUACUGGUUCAA 5‘
	5‘ CCAUUGACUUGCUGAACUU dTdT 3‘<br>3‘ dTdT GGUAACUGAACGACUUGAA 5‘
CSE gene<br>GeneID:1491	5‘ CCUUCAUAAUAGACUUCGU dTdT 3‘<br>3‘ dTdT GGAAGUAUUAUCUGAAGCA 5‘
	5‘ CACAGCAUGAGUUGGUGAA dTdT 3‘<br>3‘ dTdT GUGUCGUACUCAACCACUU 5‘
	5‘ CCGGCAAUCAUGACUCAUG dTdT 3‘<br>3‘ dTdT GGCCGUUAGUACUGAGUAC 5‘
3MPST gene<br>GeneID:4357	5‘ UCAAGACCUACGAGGACAU dTdT 3‘<br>3‘ dTdT AGUUCUGGAUGCUCCUGUA 5‘
	5‘ GCCAUCUGUUCCAGGAGAA dTdT 3‘<br>3‘ dTdT CGGUAGACAAGGUCCUCUU 5‘
	5‘ AGACGUGCCCAUCUACGAU dTdT 3‘<br>3‘ dTdT UCUGCACGGGUAGAUGCUA 5‘
siRNA Negative control	As control siRNA sequence provided by RioBio, Guangzhou.<br>Product No. siN05815122147-1-5

Supporting figures

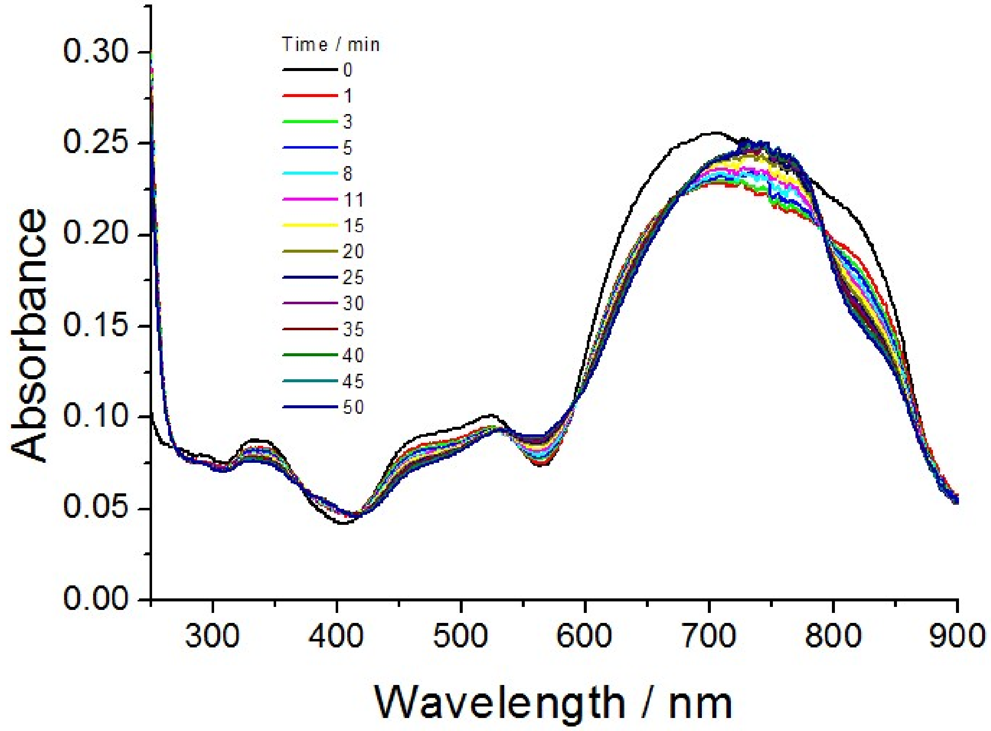

Fig. S1. Time-dependent UV-vis spectra of 10 μM probe **1** towards 200 μM H_2S in PBS buffer (50 mM, pH = 7.4, containing 10% DMSO).

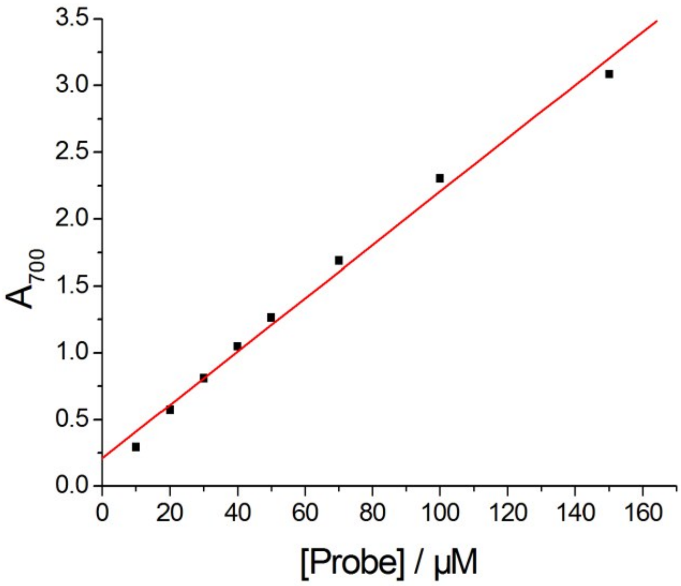

Fig. S2. The absorption at 700 nm of probe **1** of different concentrations in PBS buffer (50 mM, pH = 7.4, containing 2% DMSO). The linear relationship implied the good solubility of the probe.

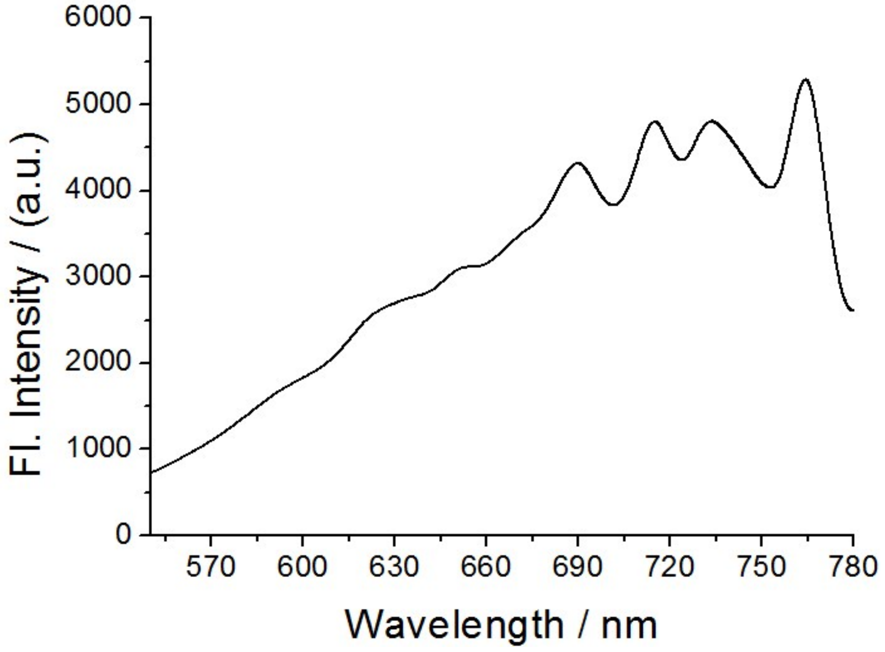

Fig. S3. The excitation spectrum (emission at 796 nm) of 10 μM probe in the presence of 100 μM H_2S in PBS buffer at 37 °C.

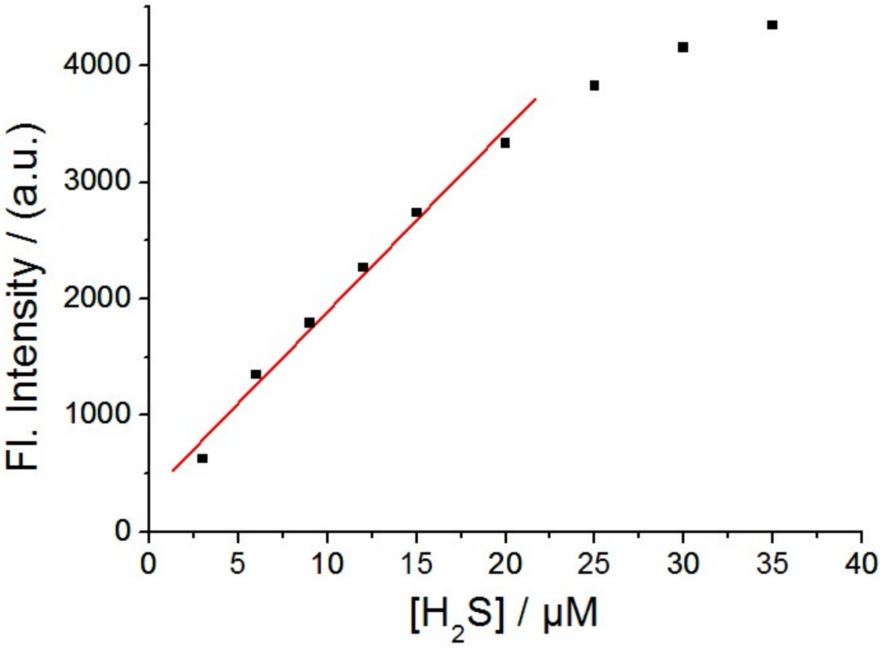

Fig. S4. The linear relationship between fluorescence intensity at 796 nm and H_2S concentration at 37 °C in PBS buffer. 10 μM probe **1** was treated with different concentration of H_2S at room temperature for 30 min.

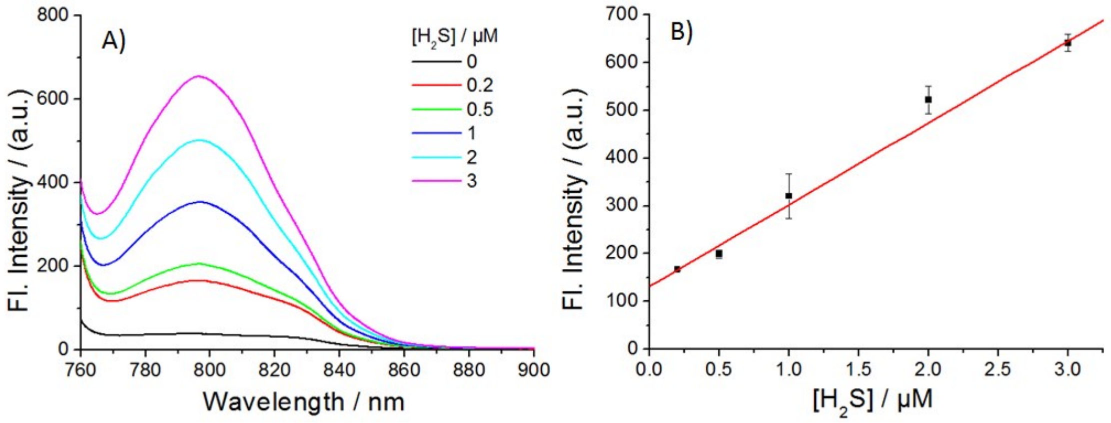

Fig. S5. Determination of detection limit based on fluorescence response of low-micromolar H_2S toward 10 µM probe **1** at 37 °C for 30 min in PBS buffer (pH 7.4). A) Fluorescence spectra of the reaction solution with 730 nm excitation. B) Linear relationship (R = 0.995) of fluorescence intensity at 796 nm versus H_2S concentration for triple titration experiments. The detection limit is determined to be 39.6 nM by using 3σ/k method, where σ (2.26) is the standard deviation of blank measurements of probe **1**; k (171.25) is the slope from red line in Fig. S5B.

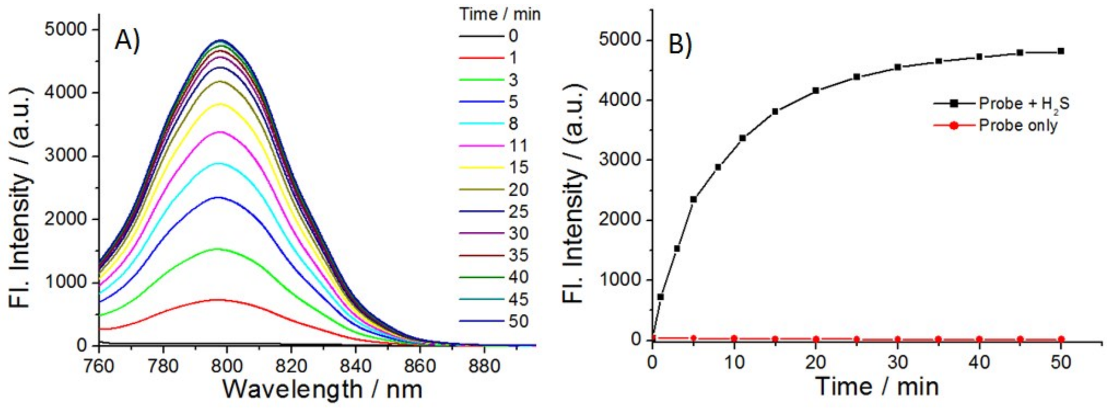

Fig. S6. A) Time-dependent fluorescence spectra (*ex*. 730 nm) of 10 µM probe **1** towards 100 µM H_2S at room temperature in PBS buffer (20 mM, pH = 7.4, containing 10% DMSO). B) Time-course fluorescence intensity at 796 nm of 10 µM probe **1** towards 100 µM H_2S or not in PBS buffer.

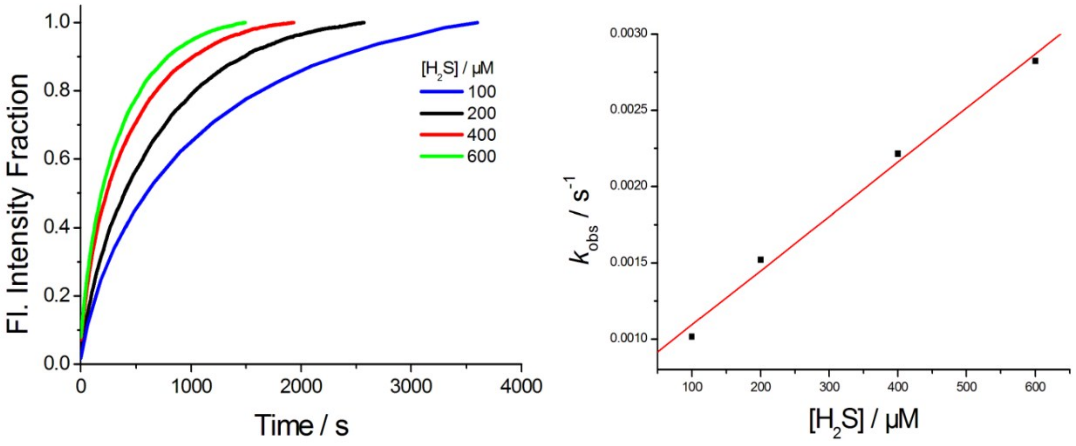

Fig. S7. Time-dependent fluorescence signal at 796 nm of 10 μM probe **1** towards different concentrations of H_2S in PBS buffer at room temperature. The pseudo-first-order rate, k_{obs} was determined by fitting the fluorescence intensity data with single exponential function. The linear fitting between k_{obs} and H_2S concentrations gives the reaction rate (k_2). The linear-relationship plot (R = 0.996) (right figure) of H_2S concentration versus calculated k_{obs} gave reaction rate of $k_2 = 3.6$ M^{-1} s^{-1}.

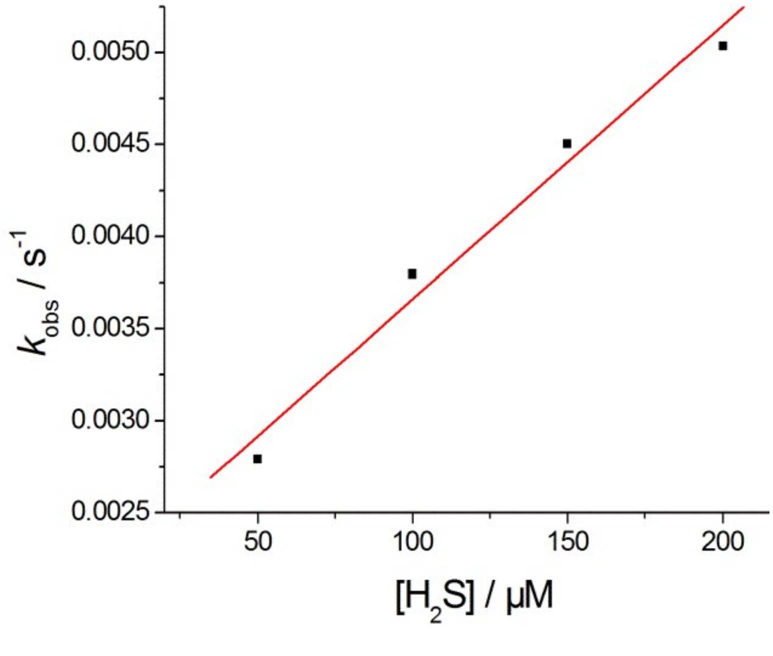

Fig. S8. The linear-relationship plot (R = 0.990) of H_2S concentration versus calculated k_{obs} of the reaction of probe **1** with H_2S at 37 ℃ gave the reaction rate 14.9 M^{-1} s^{-1}.

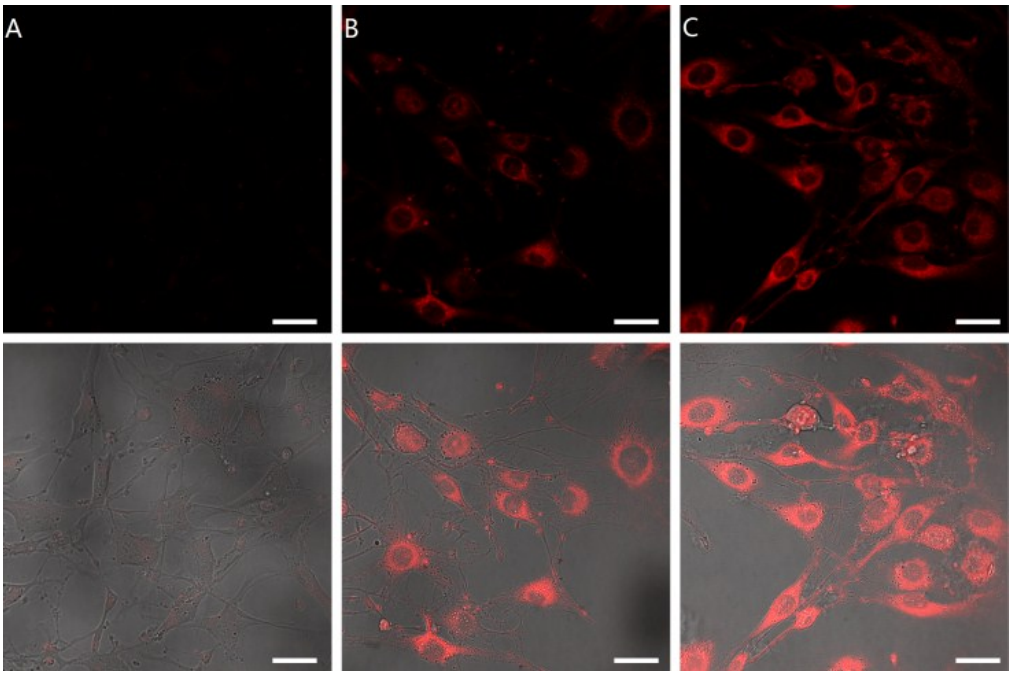

Fig. S9. Confocal microscope images of exogenous H_2S with probe **1** in living bEnd.3 cells. Cells were incubated with (A) **1** (10 μM) for 30 min, (B, C) **1** (10 μM) for 30 min and then Na_2S (150, 450 μM) for 30 min, respectively. The bright-field images are below. Scale bar, 25 μm.

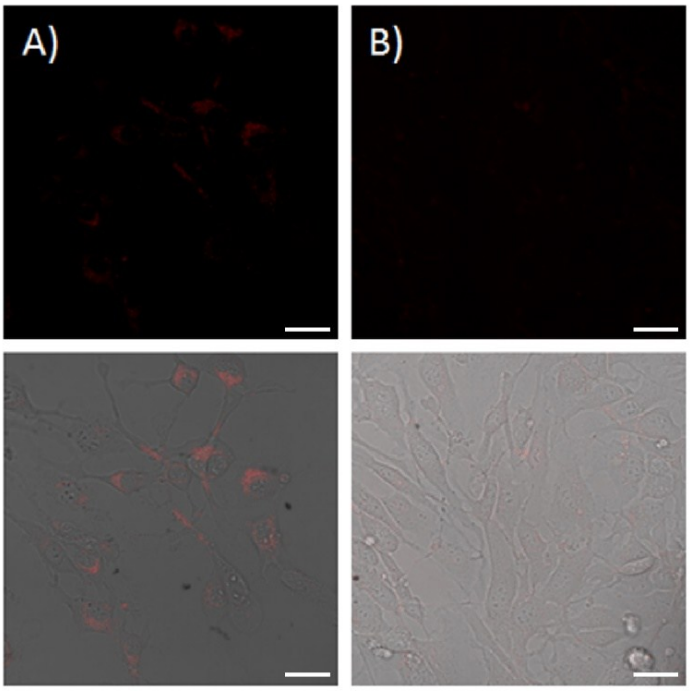

Fig. S10. Confocal microscope images of H_2S level with probe **1** in living bEnd.3 cells. Cells were incubated with (A) **1** (10 μM) for 30 min, (B) the inhibitor AOAA (200 μM) for 30 min and then **1** (10 μM) for 30 min, respectively. The bright-field images are below. Scale bar, 25 μm.

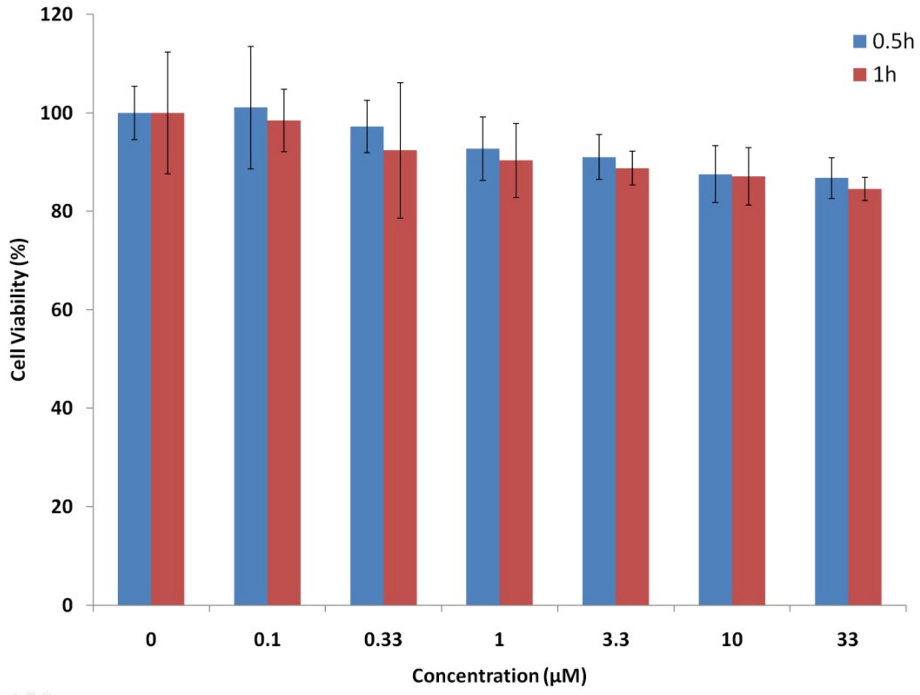

Fig. S11. Cell viabilities (%) estimated by MTT assay versus incubation concentrations of **1**.

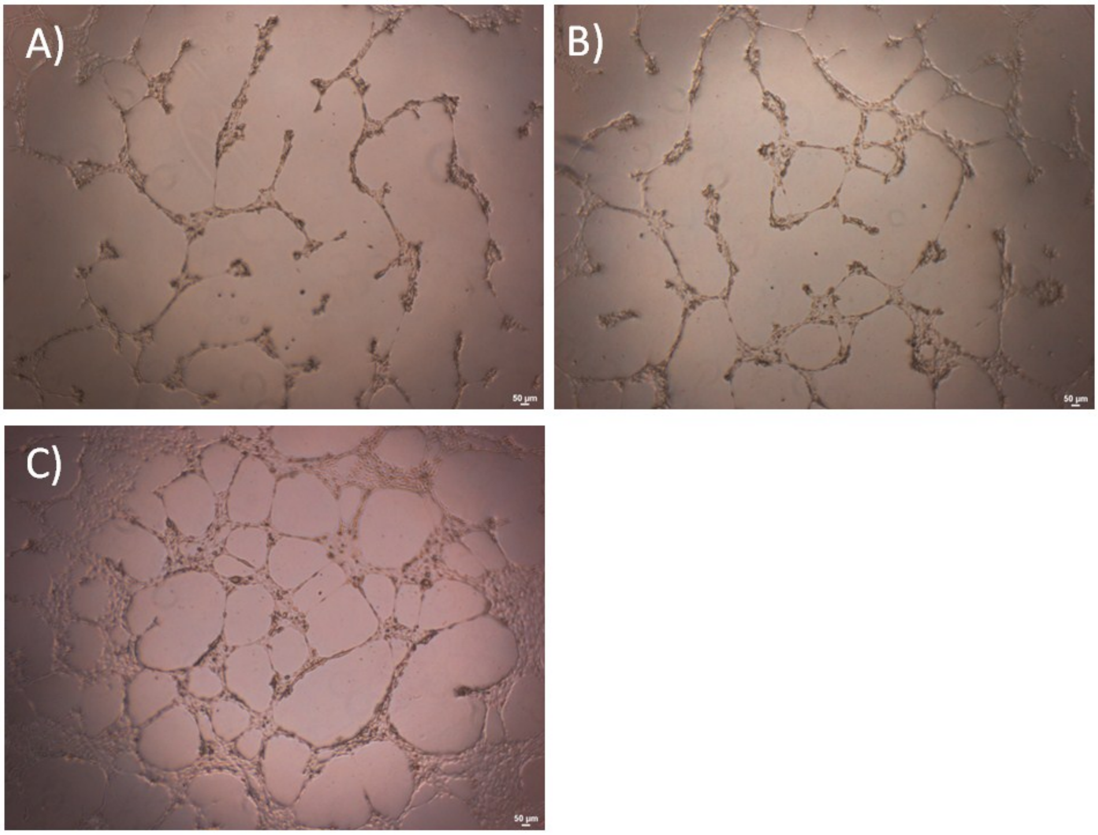

Fig. S12. Microscope images of formation of the capillary tubule structures from bEnd.3 cells in the presence of 0, 50, 150 μM D-Cys for (A-C), respectively. Scale bar, 50 μm.

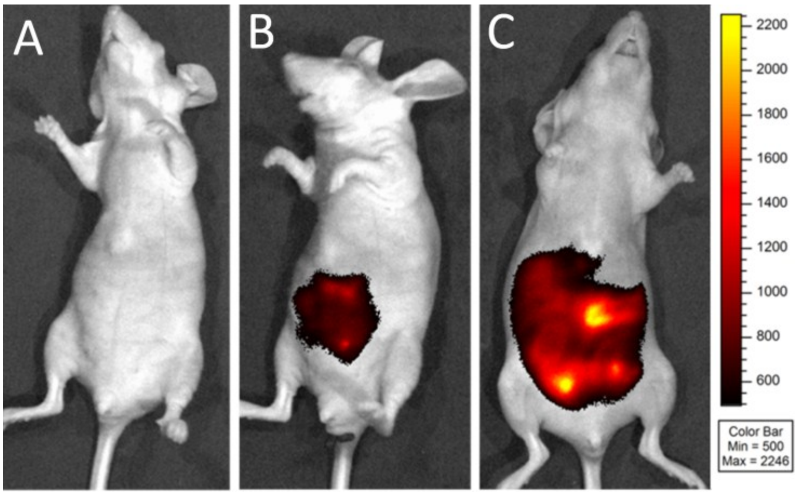

Fig. S13. Representative fluorescence images of visualizing exogenous H_2S with probe **1** in living mice. *In vivo* images of A) nude mice or B) mice with i.p. cavity injection of **1** or C) i.p. cavity injection of **1** followed by Na_2S.

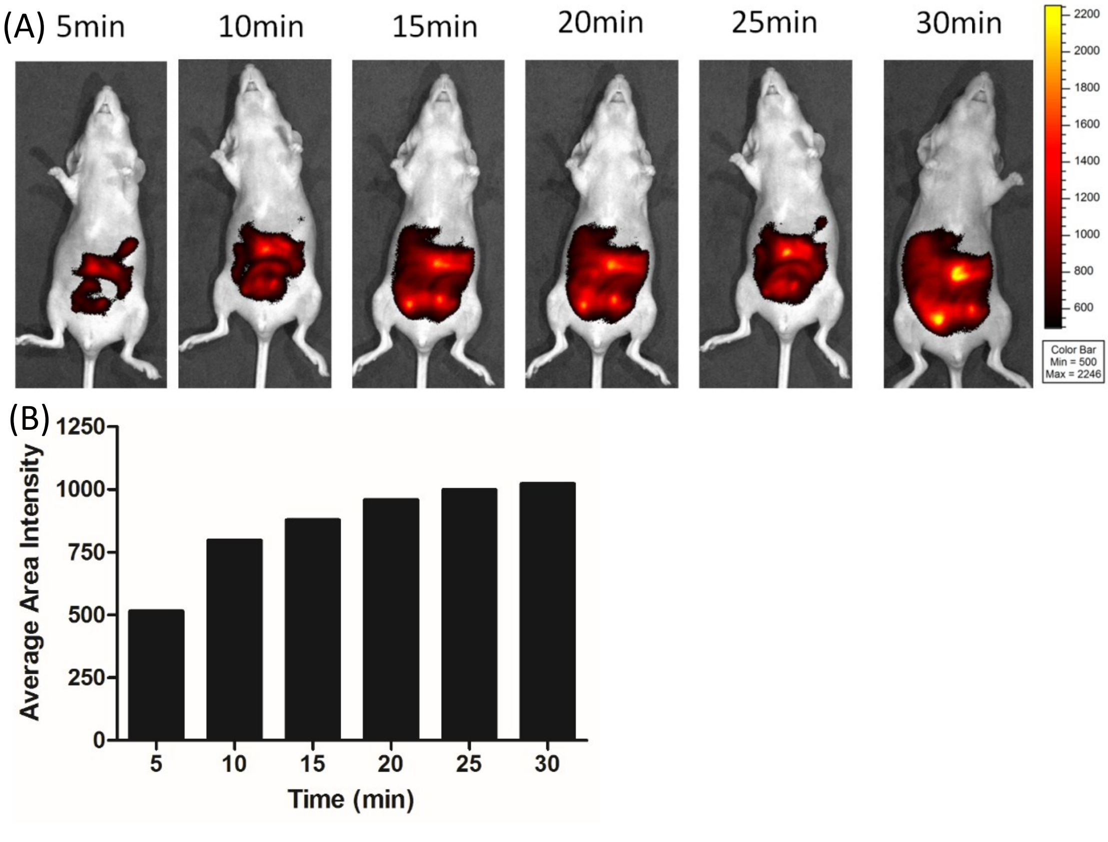

Fig. S14. Time-dependent fluorescence images of exogenous H_2S with probe **1** in living mice. (A) Mouse was i.p. cavity injection of **1** followed by Na_2S and imaged at indicated time point. (B) The average area fluorescence intensity of each image versus time. We choose a ring area that centers in the largest fluorescence and covers almost fluorescence for intensity data. The ring area for each mouse image is consistent.

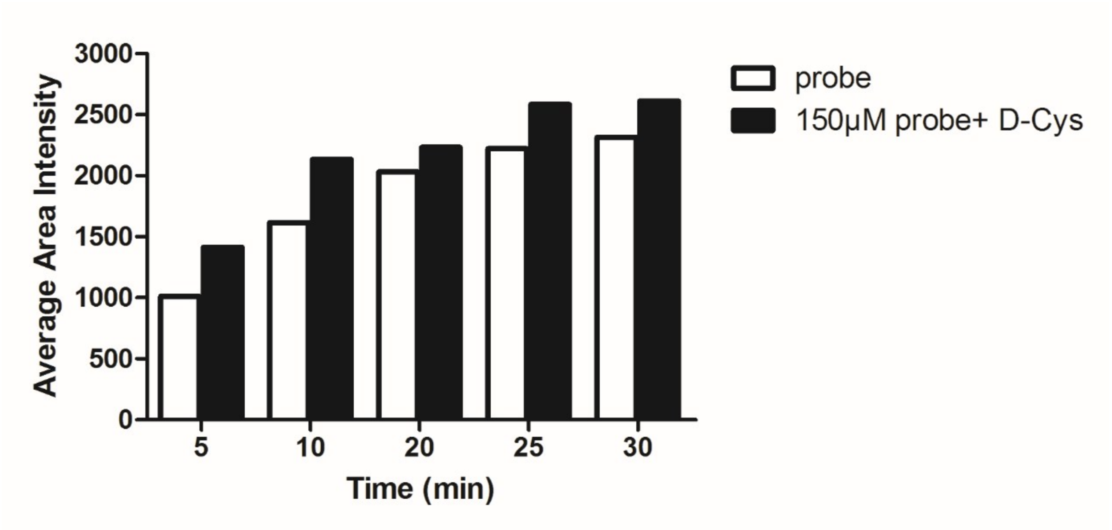

Fig. S15. The average area intensity of each image versus time in Fig. 4.

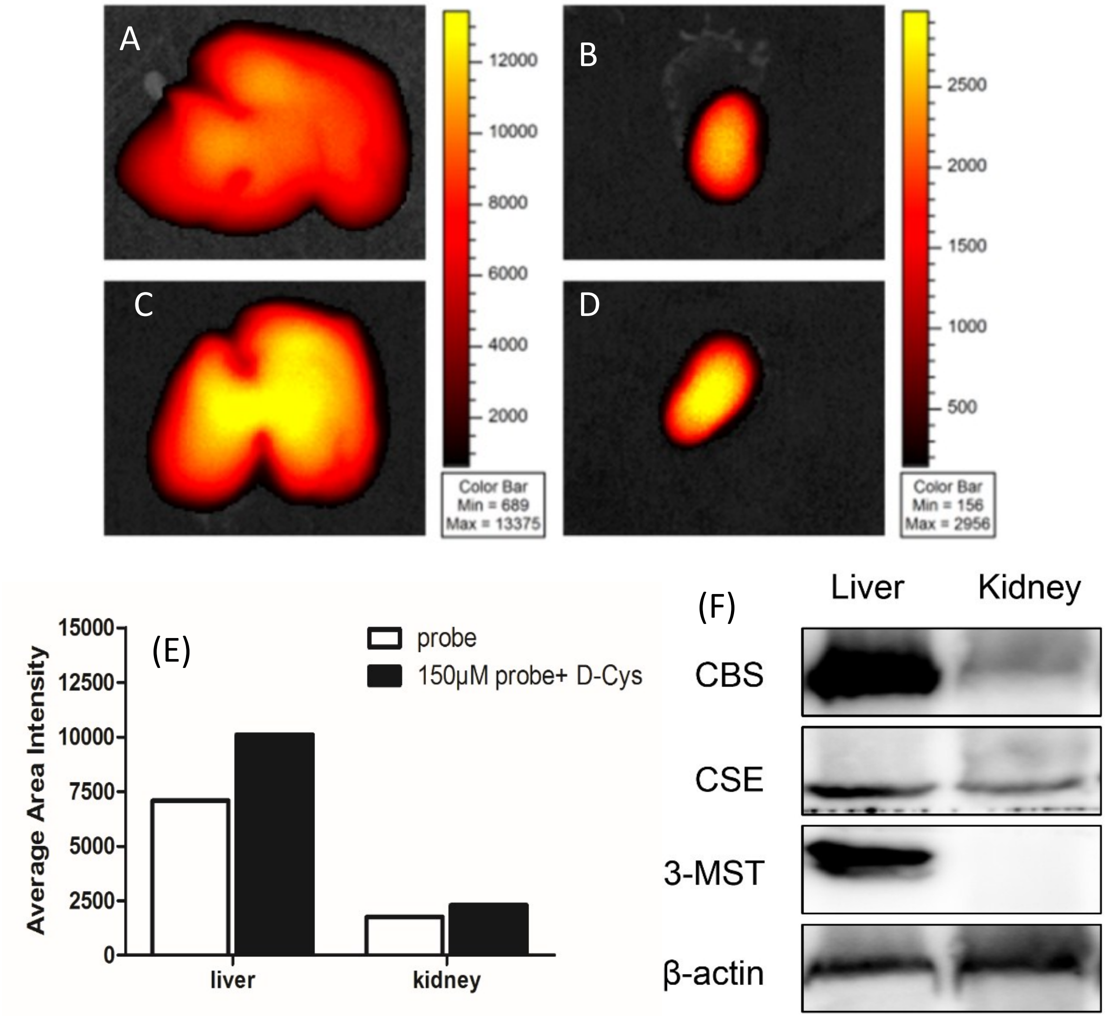

Fig. S16. *In vivo* images of liver and kidney tissues with probe **1**. (A, B) Liver and kidney from group a in Fig. 4; (C, D) liver and kidney from group b in Fig. 4. (E) The average area fluorescence intensity of tissues in A-D. (F) The expression level of H_2S-producted enzymes in mouse liver and kidney.

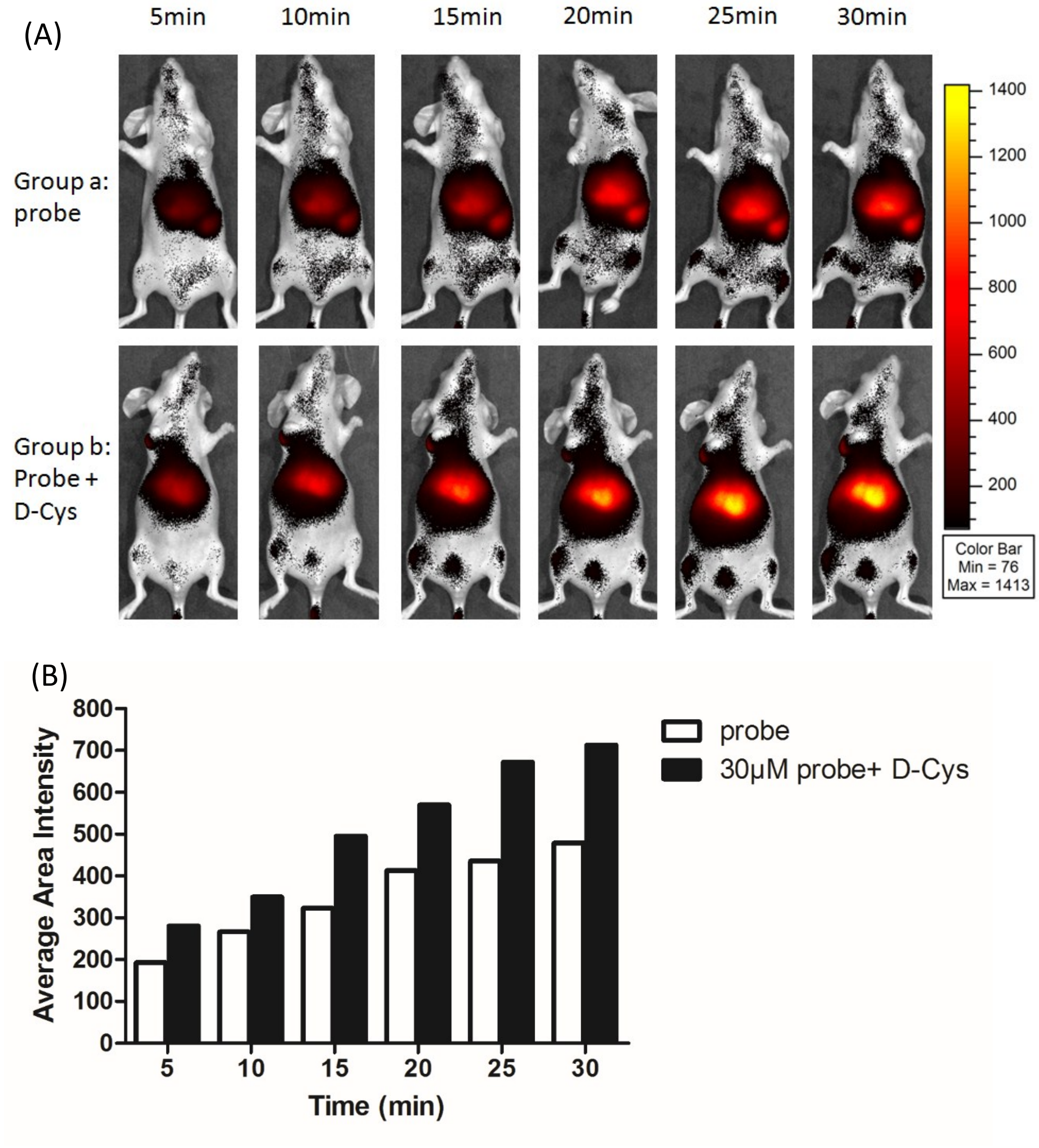

Fig. S17. Representative fluorescence images of visualizing endogenous H_2S with tail intravenous injection of probe **1** (30 μM, 200 μL) in living mice. For group a, time-dependent *in vivo* images of mouse via only injection of probe **1**; for group b, time-dependent *in vivo* images of mouse via i.p. injection of D-Cys, and after 30 min tail intravenous injection of probe **1**. (B) The average area fluorescence intensity of each image versus time.

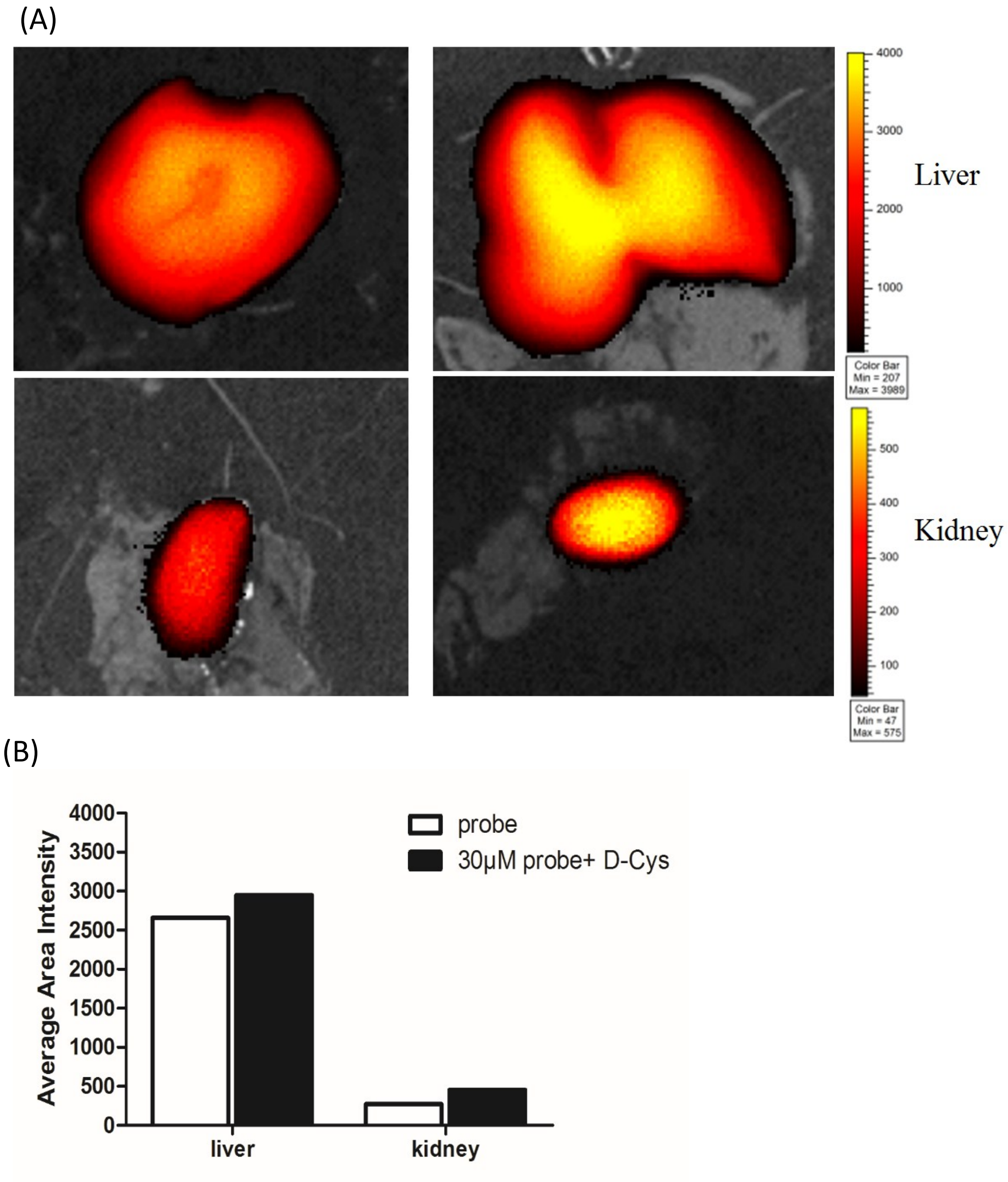

Fig. S18. *In vivo* images of liver and kindey tissues with probe **1** from Fig. S16. Left part for only probe treatment mice; right part for D-Cys treatment mice. (B) The average area fluorescence intensity of tissues.

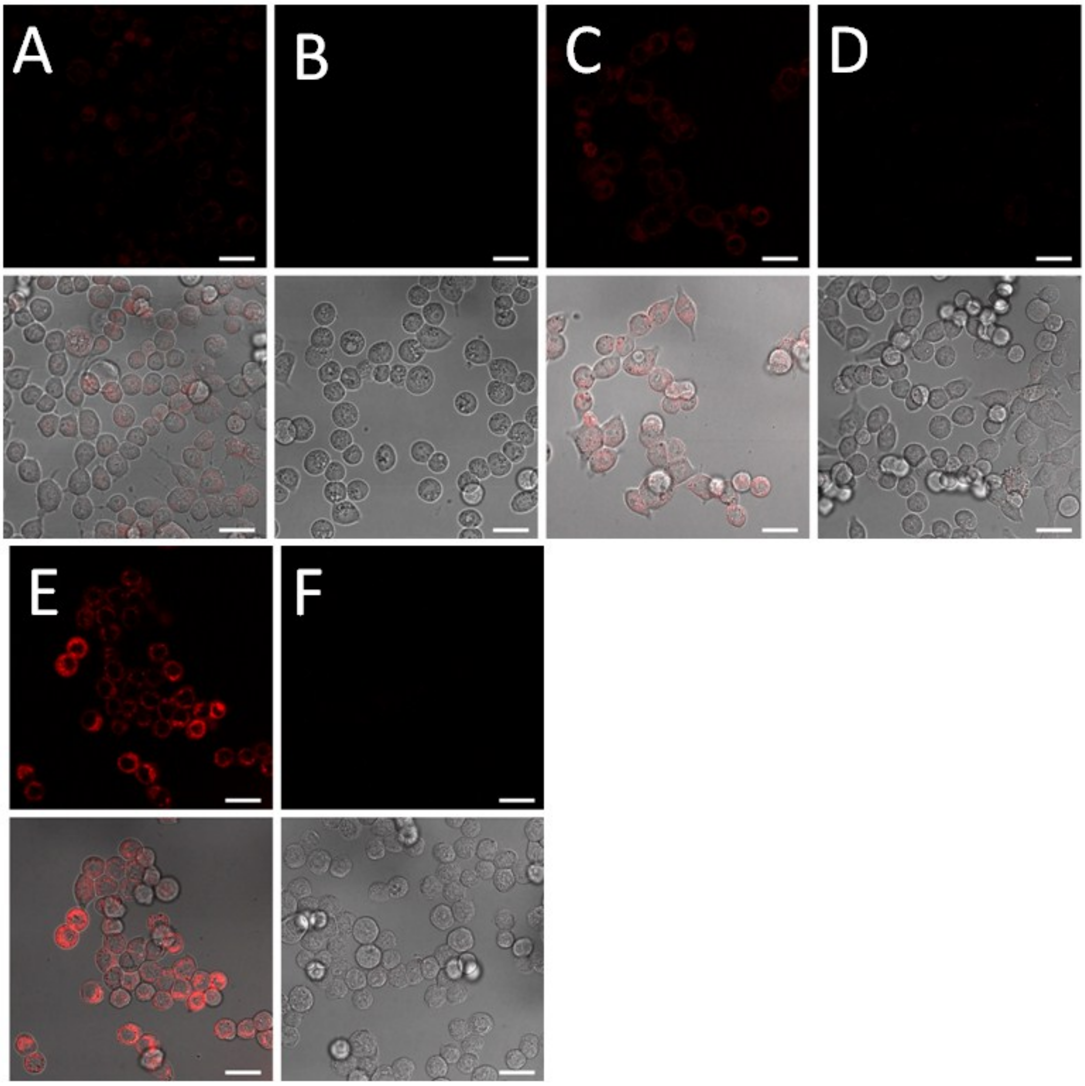

Fig. S19. Microscope fluorescence images of endogenous H_2S with probe **1** in living cancer Cells. (A) FHC cells were only incubated with 10 μM probe **1**. (B) FHC cells were pre-incubated with 1 mM $ZnCl_2$ for 30 min and then with 10 μM probe for another 30 min. (C) HCT116 cells were only incubated with 10 μM probe **1**. (D) HCT116 cells were pre-incubated with 1 mM $ZnCl_2$ for 30 min and then with 10 μM probe for another 30 min. (E) HT29 cells were only incubated with 10 μM probe **1**. (F) HT29 cells were pre-incubated with 1 mM $ZnCl_2$ for 30 min and then with 10 μM probe for another 30 min. The overlap pictures of fluorescence and bright-field images were shown as below images for each corresponding fluorescent image.

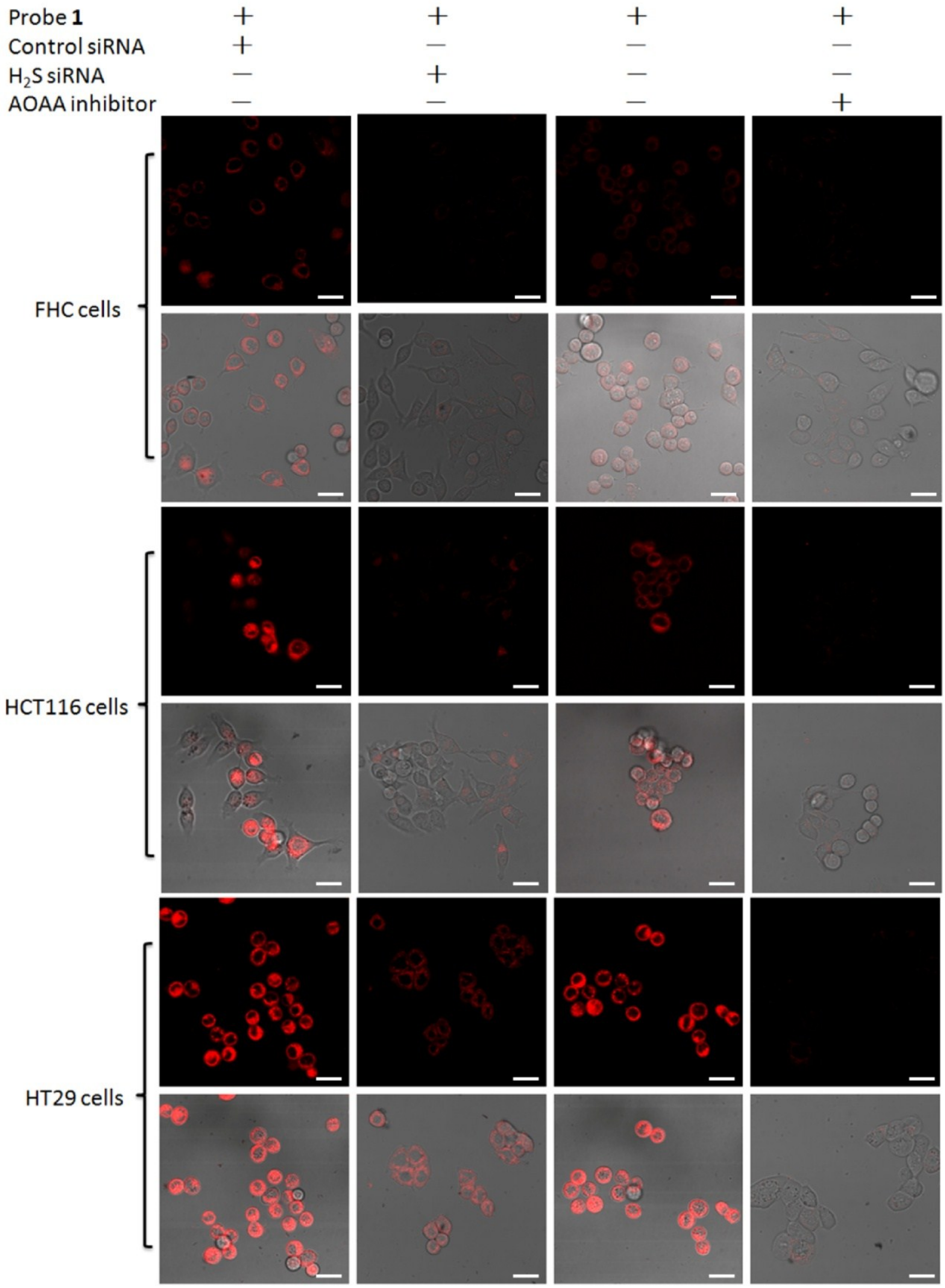

Fig. S20. Microscope fluorescence images of endogenous H_2S and its regulation via siRNA or inhibitor AOAA in living Cells. H_2S-produced enzymes were silenced using designed siRNA sequences to generate specific gene-knockdown cells. Then siRNA-silenced cells were treated with **1** (10 μM) for 30 min. For inhibiting experiments, cells were pre-incubation with AOAA (200 μM) for 30 min and then with **1** (10 μM) for 30 min. The overlap of fluorescence and bright field images are shown below. Scale bar, 25 μm. Control siRNA, cells with control siRNA sequence; H_2S siRNA, cell with siRNAs targeting H_2S-produced enzymes.

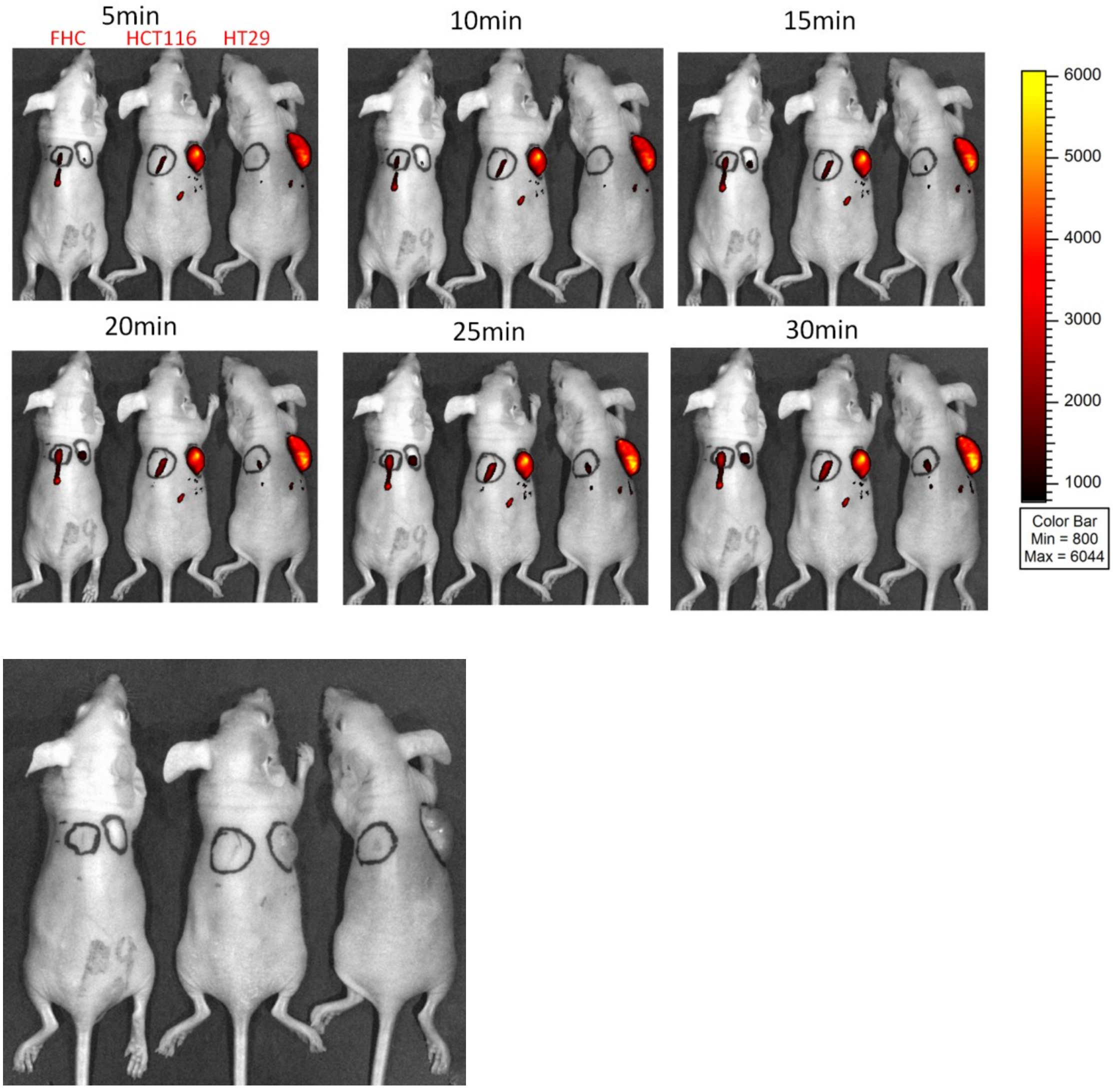

Fig. S21. Time-dependent fluorescent images of mice with skin-pop (s.p.) injection of probe **1** in Fig. 5c. The mice from left, middle, right represent FHC-, HCT116 xenograft tumor-, HT29 xenograft tumor-containing mice, respectively. The left injection position was for control purpose, while the right injection was for each grafted cells position.

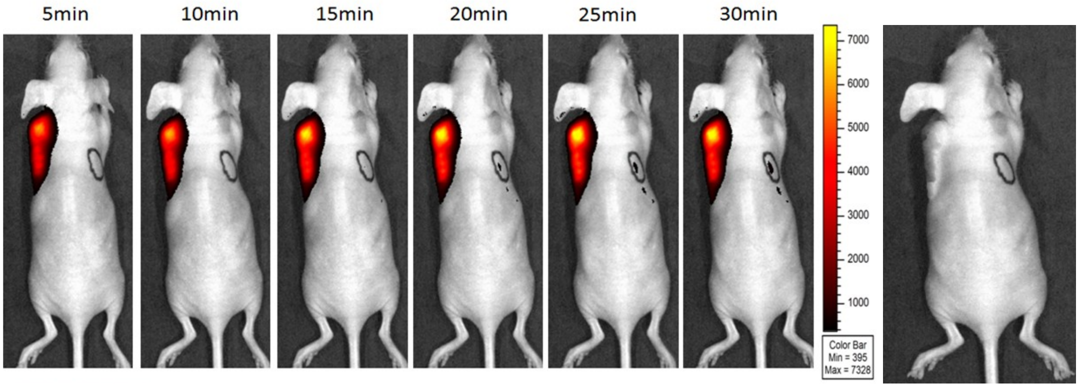

Fig. S22. Time-dependent fluorescent images of HCT116 xenograft tumor-containing mice with skin-pop (s.p.) injection of probe **1**. The right injection position was for control purpose, while the left injection was for each grafted cells position. The white light image of the mice was shown in the right.

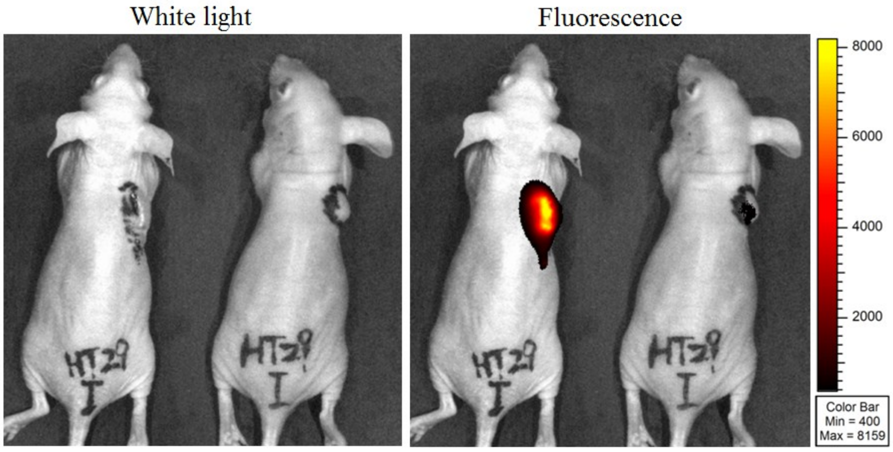

Fig. S23. *In vivo* fluorescent and white light images of HT29 xenograft tumor-containing mice with skin-pop (s.p.) injection of probe **1** in the absence (left mouse) or presence (right mouse) of inhibitor AOAA. The AOAA (at a final concentration of 200 µM for tumor volume) was pre-injected into tumor for 30 min and then the probe (at a final concentration of 10 µM for tumor volume) was injected. Images were taken 30 min after probe injection.

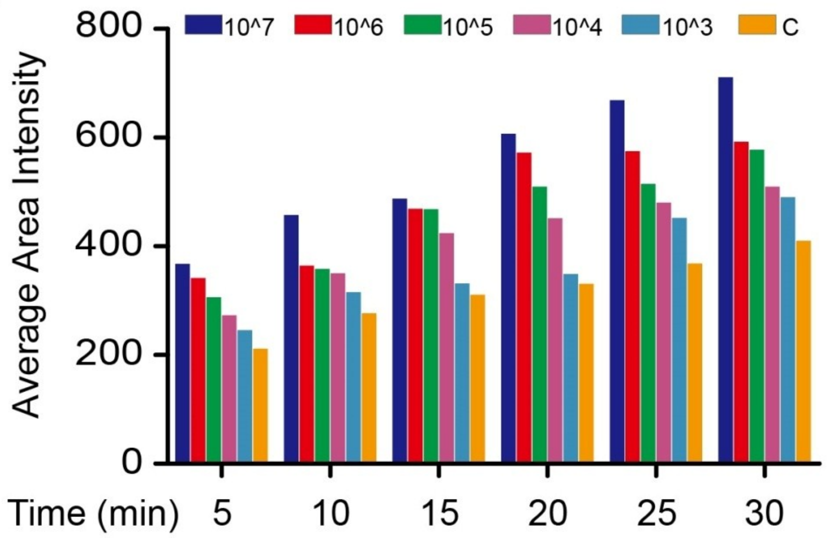

Fig. S24. Cancer cell detection limit and sensitivity of the NIR probe *in vivo*. The average area fluorescence intensity of each image in Fig. 6 versus time. C represents the control of only PBS buffer-injected position.

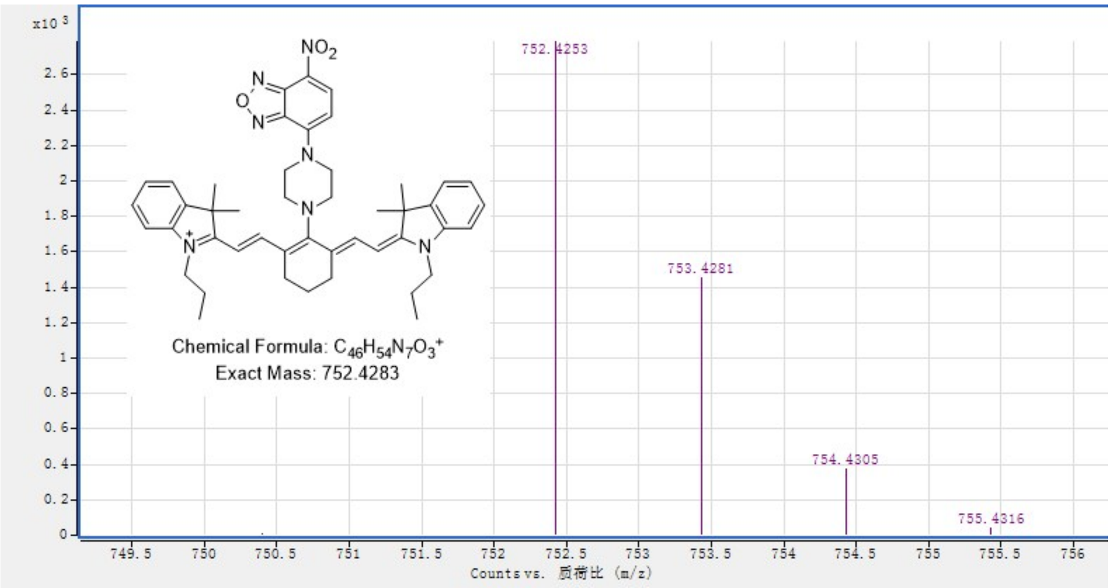

Fig. S25. HRMS spectrum of **1**.

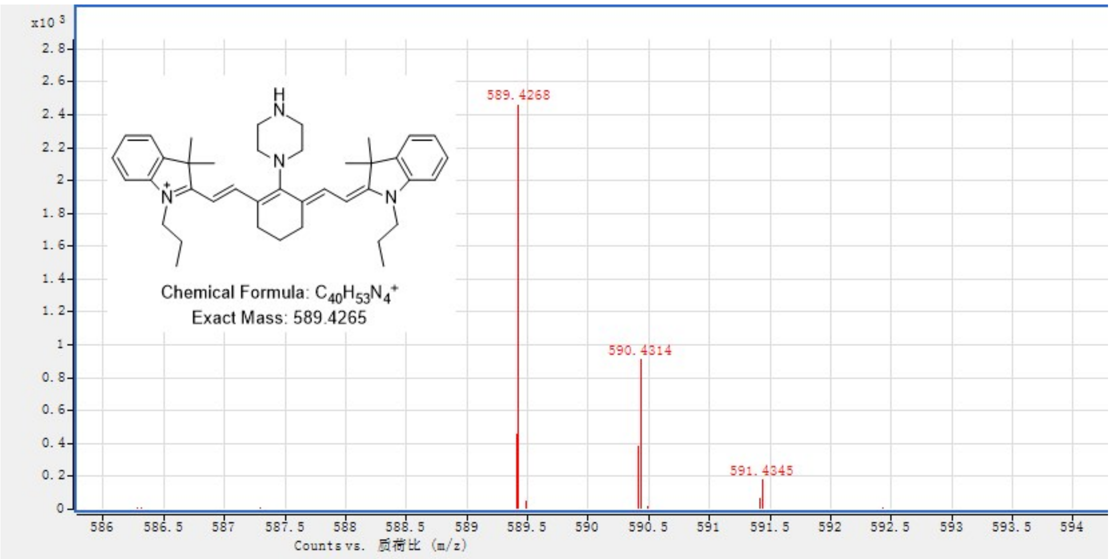

Fig. S26. HRMS spectrum of probe **1** after treatment with H_2S. 10 μL 10 mM **1** in DMSO and 10 μL 100 mM Na_2S in H_2O were co-incubated in 80 μL 50 mM PBS (pH 7.4, containing 10% DMSO) for 1 h at room temperature. Then the reaction mixture was submitted into ESI-MS without purification.

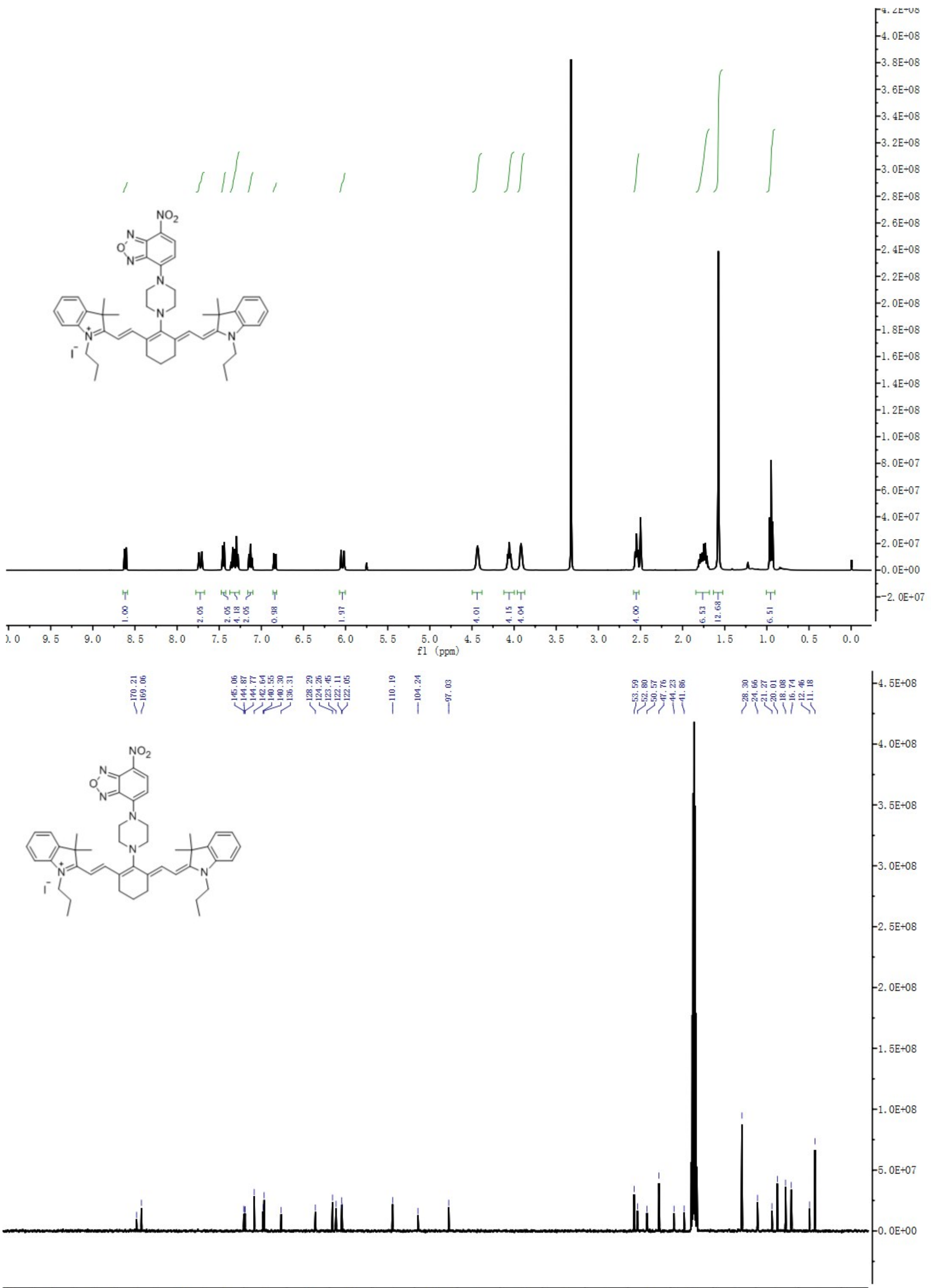

Fig. S27. ^{1}H NMR and ^{13}C NMR spectra of **1** in DMSO-d_6.
